# Peptides and Peptidomimetics as Inhibitors of Enzymes Involved in Fibrillar Collagen Degradation

**DOI:** 10.3390/ma14123217

**Published:** 2021-06-10

**Authors:** Patrycja Ledwoń, Anna Maria Papini, Paolo Rovero, Rafal Latajka

**Affiliations:** 1 Department of Bioorganic Chemistry, Faculty of Chemistry, Wroclaw University of Science and Technology, 50-370 Wroclaw, Poland; patrycja.ledwon@pwr.edu.pl; 2 Interdepartmental Research Unit of Peptide and Protein Chemistry and Biology, Department of Neurosciences, Psychology, Drug Research and Child Health-Section of Pharmaceutical Sciences and Nutraceutics, University of Florence, 50019 Sesto Fiorentino, Firenze, Italy; 3 Interdepartmental Research Unit of Peptide and Protein Chemistry and Biology, Department of Chemistry “Ugo Schiff”, University of Florence, 50019 Sesto Fiorentino, Firenze, Italy; annamaria.papini@unifi.it

**Keywords:** peptides, peptidomimetics, collagen, enzyme inhibitors, cosmeceuticals

## Abstract

Collagen fibres degradation is a complex process involving a variety of enzymes. Fibrillar collagens, namely type I, II, and III, are the most widely spread collagens in human body, e.g., they are responsible for tissue fibrillar structure and skin elasticity. Nevertheless, the hyperactivity of fibrotic process and collagen accumulation results with joints, bone, heart, lungs, kidneys or liver fibroses. Per contra, dysfunctional collagen turnover and its increased degradation leads to wound healing disruption, skin photoaging, and loss of firmness and elasticity. In this review we described the main enzymes participating in collagen degradation pathway, paying particular attention to enzymes degrading fibrillar collagen. Therefore, collagenases (MMP-1, -8, and -13), elastases, and cathepsins, together with their peptide and peptidomimetic inhibitors, are reviewed. This information, related to the design and synthesis of new inhibitors based on peptide structure, can be relevant for future research in the fields of chemistry, biology, medicine, and cosmeceuticals.

## 1. Introduction

Collagen is one of the most essential proteins in the human body and a major component of the extracellular matrix (ECM) [1]. There are 28 classified collagen types, of which collagen type I forms around 85%, with up to 15% formed by type III [2,3]. Type I composes mainly the tissue fibrillar structure, while type III functions as the linchpin of type I fibres and supports skin elasticity. Collagen, in general, represents approximately one-third of all human body proteins, being the most abundant protein-like body component [4]. On the basis of molecular structure, collagen is classified into three morphological forms, namely fibrillar, non-fibrillar, and network-forming collagens [5]. 

Three polypeptide collagen molecules spontaneously form a helical structure, so-called tropocollagen. A single chain (α chain) has usually the length of about 1000 residues [6]. Tropocollagen is stabilized by covalent bonds and hydrogen interactions between hydroxyproline (HyP) and hydroxylysine (HyK) [2]. It was observed that tropocollagen superhelix is visibly more constrained, with the rise per residue value of 0.29 nm comparing to 0.36 nm for common proteins [7]. This feature makes it exceptionally resistant to the widespread proteolytic digestion, making collagen susceptible only to a few enzymes [8]. Remarkable regularity of repeated amino acid triad, Gly-X-Y (where X—proline, Pro; Y—hydroxyproline, HyP), provides a variety of beneficial properties—for instance, stability and resistance to distortion. Glycine replacement by larger and more branched residues provokes inaccurate folding and decreases helix resistance to external conditions, i.e., temperature [7].

The essential role played by collagen in the human body has a relevant influence on many physiological, as well as pathological states. Collagen turnover is the complex and interconnected process of collagen biosynthesis and degradation. In physiological states, the degradative pathway acts as a regulator of collagen deposition, thus preventing various fibrotic disorders. Moreover, a proper intracellular collagen degradation prevents the secretion of defective sequences [9]. Accordingly, the control of collagen homeostasis is one of the major factors in bleeding syndromes therapies [10], while hyperactivity of fibrotic process and collagen accumulation results with joints, bone, heart, lungs, kidneys or liver fibroses [4,11,12]. Per contra, dysfunctional collagen turnover and its increased degradation lead to wound healing disruption, skin photoaging, and loss of skin firmness and elasticity [13]. The latter aspects are particularly relevant in the field of aesthetic medicine and cosmetics, to which we will pay special attention.

In this review we will briefly describe the collagen turnover process in relevant cells and tissues, indicating the main degradation pathways in particular and underlining the key enzymes involved in these mechanisms. We will review peptide-based inhibitors of collagenases, elastase, and cathepsin, mentioning also some relevant non-peptide compounds. The final aim is to give a valuable starting point for future research, providing chemical and biological basis for the design and the development of new peptide-based inhibitors, potentially applicable as cosmeceuticals.

## 2. Collagen Turnover in Cells

In this section, we briefly describe the collagen turnover process in relevant cells, in order to clarify its biosynthesis and degradation pathways. This complex process has been discussed in details in the following reviews: Rodriguez-Feo et al. [14], Gelse et al. [15], Smith and Rennie [4], Sprangers and Everts [12], together with the review by Vannella and Wynn, depicting the wound healing process and tissue remodelling [16].

Each fibrillar-type collagen is synthesised in fibroblasts in the form of procollagen, consisting of two N- and C-terminal propeptide domains [12,15]. There is a spectrum of already known collagen synthesis stimulators, such as Transforming Growth Factor beta (TGF-β), Endothelin-1 (ET-1), Angiotensin-II (Ang II), Platelet-Derived Growth Factor B (PDGF-BB), Interleukin-1 (IL-1), Aldosterone, Interferon g (IFN-g), Basic Fibroblast Growth Factor (bFGF) and Nitric Oxide (NO) [14]. As indicated previously, proline residues play a pivotal role in the collagen structure formation. At this point, Pro undergoes the hydroxylation by prolyl-3- and -4-hydroxylases, together with lysyl-hydroxylase acting on Lys [1]. Then, fibrillar procollagens are secreted into the extracellular space. Afterwards, terminal noncollagenous fragments are removed via enzymatic cascade, thus enabling the spontaneous formation of fibrils. This process, called fibrillogenesis, is regulated by collagen type V and this assembling is favoured by covalent cross-linking between lysine side chains [1,12].

As far as degradation is concerned, the tenor of collagen disruption is strictly connected to its type. Mainly, there are two recognized categories of these pathways: extracellular and intracellular. Extracellular degradation includes the cleavage of mature collagen, while the intracellular mechanism degrades the procollagen chains, before their assembling into procollagen molecules [17]. These processes prevent the formation of defective collagen and its incorporation into the extracellular matrix.

The extracellular pathway is due to secreted proteolytic enzymes, capable to digest collagen fibres. Various enzyme classes participate in this process, i.e., matrix metalloproteases (MMPs), serine proteases, active at neutral pH, as well as cysteine, aspartate, and threonine proteases, requiring mainly acidic environment [18]. MMPs target a broad range of proteins, depending on the enzyme type. For example, stromelysins (MMP-3 and -10) act preferentially on proteoglycans, fibronectin, and laminin [18]. Other MMPs, such as gelatinases, cleaves already denaturated collagen only. ECM modulation is affected also by the so-called A Disintegrin and Metalloproteinase (ADAM) and ADAM with Thrombospondin Motifs (ADAMTS) family [18,19]. The extracellular degradation process and the ECM dynamics, have been reviewed by Lu et al. in 2011 [18].

The intracellular mechanism follows the internalization of collagen fragments, previously partially degraded in the ECM [12]. The kinetics of this process was found to be very rapid [20,21]. Time and percentage of degradation depends on the tissue type, revealing significant differences between, e.g., skin and heart. Two intracellular localizations of collagen degradation have been proposed: one within the lysosome and the second within the cisternae (in the endoplasmic reticulum or Golgi apparatus).

**Figure 1:**
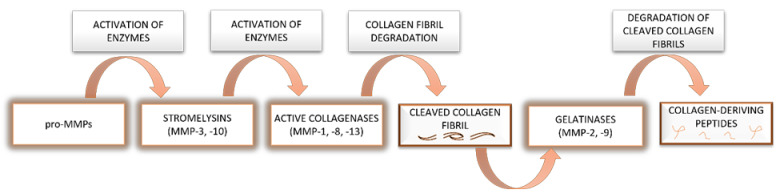
Collagen degradation pathway involving various MMPs [26].

In this review, we will put a particular attention on the extracellular breakdown pathway, describing the most substantial enzymes involved in this process. Table 1 presents a list of enzymes participating in the degradation, with reference to the relevant collagen class. Figure 1 illustrates the influence of various MMPs on extracellular collagen breakdown. Other factors indirectly stimulating collagen disruption are the following [13]: (1) ultraviolet radiation, inducing MMPs and enhancing their proteolytic action; (2) age, due to the overactive secretion of proteases observed in elder skin, together with incorrect (or diminished) fibroblasts activity [2]; (3) sex, in view of an inhibitory effect of oestrogen on collagen synthesis [22]. Two interesting papers, describing the influence of menstrual cycle on collagen synthesis, were published in 2006 and 2007. These investigations did not show any significant change between luteal or follicular phases. However, some differences between male and female collagen synthesis and remodelling were observed [23,24,25].

In this review we will focus on enzymes degrading mainly fibrillar collagen, leading to loss of skin elasticity and skin aging in general, due to their cosmeceutical interest, i.e., collagenases (MMP-1, 8, and 13), elastase, and cathepsins, together with their inhibitors. It must be highlighted that there are two pathways of enzyme activity control—direct (inhibition) and indirect (up or downregulation of enzymes expression) [42]. The following paper will consider only inhibitors directly affecting enzymes activity.

### 2.1. Methods of Collagen Turnover Measurements

**Scheme 1:**
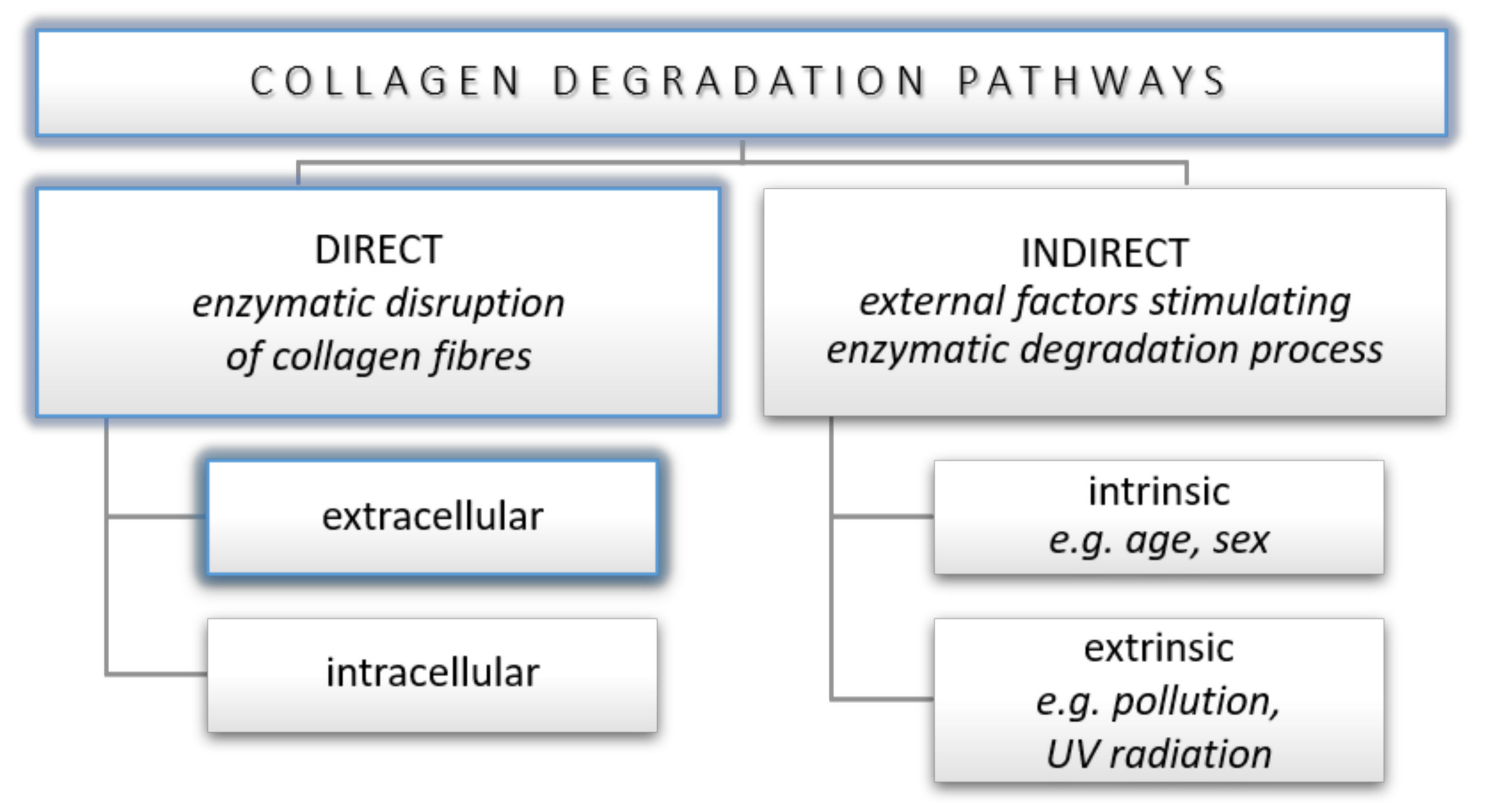
Collagen degradation pathways in cells. The focus of this review, enzymes participating in extracellular degradation, was highlighted in blue.

**Scheme 2:**
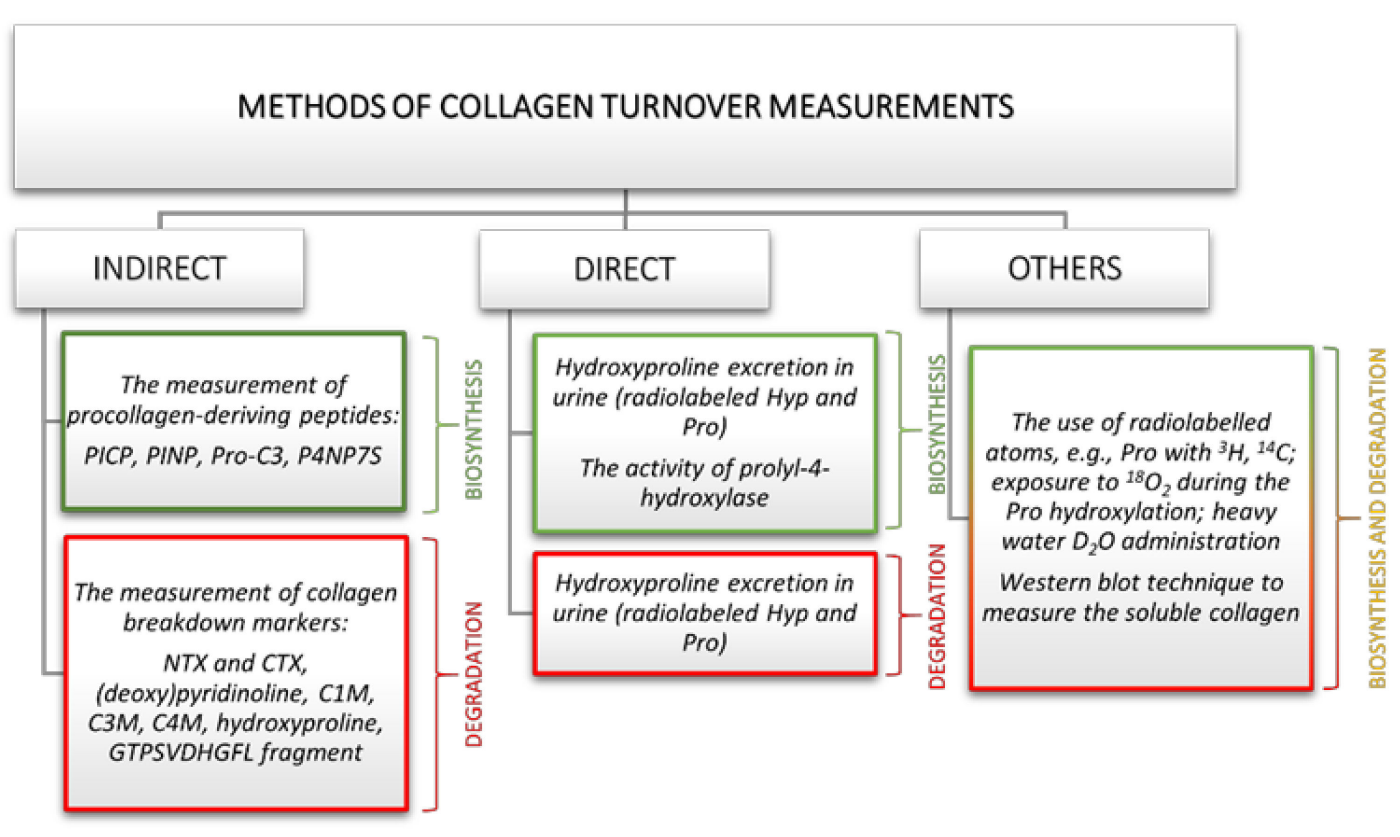
Methods of collagen turnover measurements discussed in this review. All abbreviations are explained in the text above.

As stated above, there are two interrelated pathways, contributing to collagen degradation in cells: intracellular (based on lysosomal digestion) and extracellular (initially engaging collagenases and later on also other proteases) (Scheme 1) [4]. Due to their correlated activity, cellular collagen turnover measurement, including both biosynthesis and degradation, became a challenge. Nevertheless, there are a few methods directly and indirectly evaluating this factor. These discussed in our review are summarized in Scheme 2.

#### 2.1.1. Indirect Methods

Collagen synthesis can be followed by the rate of appearance of specific peptides, deriving from procollagen: (1) PICP, C-terminal pro-peptide from collagen type I; (2) PINP, N-terminal pro-peptide from collagen type I; (3) Pro-C3, internal epitope in the N-terminal pro-peptide from collagen type I; (4) P4NP7S, internal epitope in type IV collagen 7S domain [4,25].

Degradation process can be monitored by the measurement of collagen breakdown markers, such as: (1) NTX and CTX, crosslinked telopeptides of type I collagen; (2) (deoxy)pyridinoline, pyridinium crosslinkers of type I collagen; (3) C1M, neo-epitope of MMP-2,9,13- mediated collagen type I degradation; (4) C3M, neo-epitope of MMP-9- mediated collagen type III degradation; (5) C4M, neo-epitope of MMP-2, -9, -12-mediated collagen type IV (α1 chain) degradation [4,25]. In order to estimate collagen degradation products, Nielsen et al. measured the CTX-II, CTX-I, and hydroxyproline content in protein extract from joints [43]. Therefore, they indicated “an assessment of collagen degradation epitopes as a quantitative measure of cartilage damage”.

Veidal et al. in 2011 described the applicability of ELISA assay in measuring a degradation fragment specific to the type IV collagen, namely GTPSVDHGFL [44]. This neoepitope is generated during MMP-induced degradation of collagen and was associated with liver fibrosis in animal models.

Smith and Rennie, who listed in their review several of the above-mentioned methods, put attention on the applicability of indirect measurements [4], underlining that using these methods also some procollagen can be measured. Therefore, they are semi-qualitative and cannot define a precise content of mature collagen in a probe. 

#### 2.1.2. Direct Methods

One collagen monomer contains approximately 13% of hydroxyproline residues. Starting from this observation, Hyp excretion in urine has been used as a collagen degradation indicator. In 2005, McAnulty proposed the application of radiolabelled Hyp and Pro for in vitro and in vivo determination of collagen synthesis and degradation [45]. Moreover, the activity of prolyl-4-hydroxylase can be evaluated as an index of collagen synthesis. This enzyme catalyses the proline hydroxylation, one of the initial steps in collagen post-translational processing [46]. The reaction takes place exclusively during the collagen biosynthesis, therefore can be employed for the above-mentioned purpose. Gorres and Raines in 2010 described for the first time a relatively simple and direct assay for prolyl-4-hydroxylase, based on a detection of the Pro-containing substrate turnover [47]. 

#### 2.1.3. Others

Widely described methods of collagen turnover monitoring include the use of radiolabelled atoms, e.g., Pro with ^3^H, ^14^C; exposure to ^18^O_2_ during the Pro hydroxylation; heavy water D_2_O administration (followed by deuterium incorporation into Ala and Pro).

In 2018, Cipriani et al. described a quantitative detection of soluble collagen by western blot technique, similar to the protocol previously published by Poobalarahi et al. [27,48]. Briefly, fibroblast cell cultures were treated with trypsin in order to analyse the intracellular forms of collagen. The presence and intensity of bands corresponding to procollagen were then observed in cell lysates.

## 3. Inhibitors of Collagen-Degrading Enzymes

Fibrillar collagen is responsible for skin elasticity, firmness, and overall wellness and good appearance. In the field of cosmeceuticals, being on the border between traditional cosmetics and drug-like products, the claimed effect of a given cosmetic product should be connected with proven efficacy and activity of its active ingredient(s) [49]. Therefore, collagen degradation inhibitors and collagen biosynthesis enhancers are highly requested and broadly investigated in the recent years. Among other classes of active ingredients, peptides play an important role in these fields, due to their high specificity, low toxicity, relatively easy synthesis, and applicability as carriers for other molecules [50]. However, peptides in some cases get degraded very quickly on the skin surface, or are not able to penetrate sufficiently into the skin layers [51]. In the aim to enhance peptides stability and efficacy as bioactive ingredients, the use of peptidomimetics is increasingly considered as a viable alternative to unmodified peptides.

**Figure 2:**
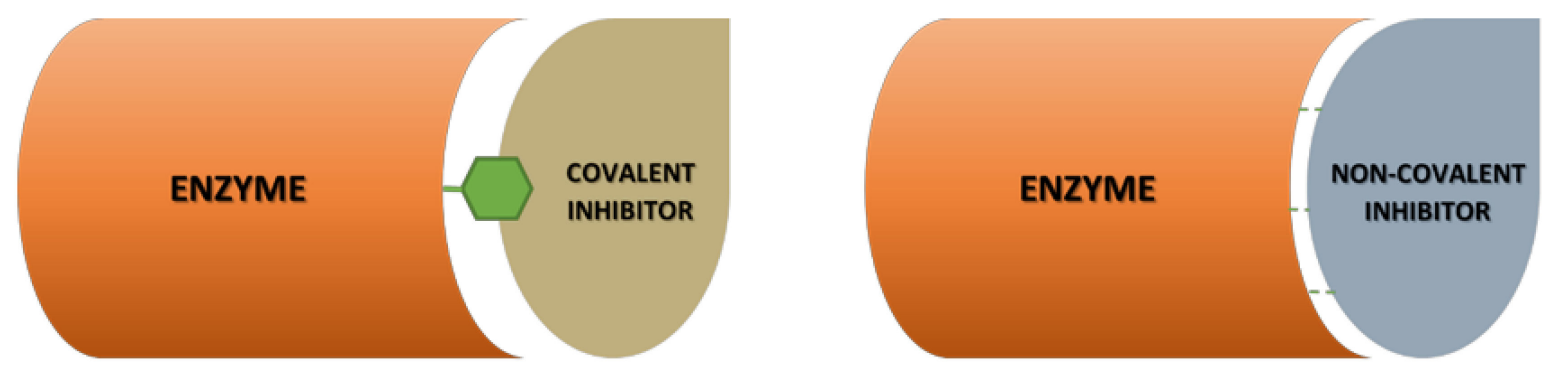
Illustration of two binding pathways in major inhibition mechanisms: covalent (left) and non-covalent (right) [53].

In general, two mechanisms of inhibition can be identified: covalent and non-covalent binding pathways [52] (Figure 2). The covalent mechanism is based on specific binding to the target protein, with formation of a permanent complex with the enzyme. On the other hand, non-covalent inhibition assumes the semi-permanent interaction between enzyme and inhibitor, through cyclic binding, unbinding, and rebinding [53]. Comparing both these pathways, covalent inhibition is advantageous over the non-covalent one. The presence of the inhibitor warhead allows the formation of particular interactions that leads to the development of highly specific inhibitors. Cysteine, serine, threonine or lysine residues participate in covalent bonds formation due to their nucleophilic character; hydroxyl, epoxy or carbonyl moiety in the ligand structure are usually responsible for the arrangement of these covalent adducts [54].

### 3.1. Collagenases

MMPs inhibitors (so-called MMPIs) have been investigated for many years, with the first publications from 1980. The first synthetically obtained inhibitors were characterized by functional groups able to coordinate a zinc(II) ion in MMPs active site [55]. Relatedly, four classes of zinc MMPIs can be designated: (1) hydroxamates (CONH-O^−^); (2) carboxylates (COO^−^); (3) thiolates (S^−^); and (4) phosphinyls (PO^2−^) [56]. Recently, some commercially developed MMPIs were submitted to clinical trials, such as batimastat, marimastat, and others [55]. These two were then approved as drugs [57]. Despite many efforts in this field, selective inhibitors of specific MMPs have not been found yet. This is probably due to the complicated mechanism of MMPs proteolytic activity, and mutual interactions of many factors, leading together to the inhibition process [56]. This mechanism has been studied for years, among others by Moroder and his group, which reported their results in 2000 [58]. They examined the substrate specificity and preferences of collagenases and gelatinases, using synthetic peptides derived from collagen. It was clearly indicated that the inhibitory activity of both classes has different basis, but since the activity of all MMPs is zinc-dependent, compounds containing chelating groups are generally good, but weakly selective MMPIs. More detailed structural data and widely described catalytic activity of metalloproteases, are described in two papers by Bode and Maskos [59,60].

Studies focused on structure-activity relationship (SAR) of MMP-13 inhibitors were described by Roush et al., providing relevant indications for further developments [61,62]. In addition, both papers include the evaluation of amino acids derivatives, which will be further described in Section 3.1.4.

#### 3.1.1. Peptide Hydroxamates

To the best of our knowledge, the very first paper describing the hydroxamate group (CONH-O^−^) as an efficient inhibitor of collagenases was published in 1986 by Moore and Spilburg [63]. They studied the influence on skin fibroblasts collagenases activity of various peptide sequences, with diverse C-terminal groups (amide, carboxylate, aldehyde, and hydroxamate). Comparing the percentage of inhibition among amastatin, captopril, phosphoramidon, and zincov (standard metalloproteases inhibitors; structures are reported in Table 2) in 0.5 mM concentration, it was proven that only zincov was able to decrease the collagenases activity. Afterwards, a few sequences of hydroxamic acid derivatives were synthesised, in order to evaluate the influence of their length on inhibition efficacy. The lowest IC_50_ value was reported for Z-Pro-Leu-Gly-NHOH (0.04 mM), while shortening the sequence yielded less-active inhibitors, achieving IC_50_ = 40 mM for acetohydroxamic acid. It was deduced that collagenases distinguished the sequence including Pro due to its similarity to the cleavage site in native collagen, recognized by the enzyme. Finally, to determine the role of hydroxamate in the inhibition mechanism, Z-Pro-Leu-Gly-X derivatives were investigated (where X indicated hydroxamate, carboxylate, aldehyde, and amide modification on C-terminus). The results obtained for the peptide sequence with unmodified glycine residue showed the importance of this sequence in the inhibition mechanism. The C-terminally merged hydroxamate led to satisfactory results (C_M_ = 5.3 mM and 15% of inhibition for carboxylate; C_M_ = 0.115 mM and 70% of inhibition for hydroxamate derivative).

In 1992, Grobelny et al. confirmed the applicability of peptide hydroxamic acids as collagenase inhibitors [64]. HONHCOCH_2_CH(*i*-Bu)CO-Trp-NHMe sequence isomers (L,L-dipeptide **6A** (*R*,*S*) and D,L-dipeptide **6B** (*S*,*S*)) were designed and synthesised to evaluate the efficacy against human skin fibroblast collagenase, thermolysin and *Pseudomonas aeruginosa* elastase. the structural difference between **6A** and **6B** consists in the CH_2_CH(*i*-Bu)CO α-carbon configuration, thus **6A** is in the L-, and **6B** in the D-configuration. Surprisingly, these diastereomers distinguished very evidently the discrepancy between inhibitory preferences among human (collagenase) and bacterial (thermolysin and elastase) enzymes. It was confirmed that the **6A** inhibits collagenase with *K*_i_ = 0.4 nM; in the case of **6B**, this value was of 200 nM. On the contrary, bacterial enzymes were inhibited with comparable potency by both **6A** (*K*_i_ = 20 nM for each protein) and **6B** (*K*_i_ = 7 nM and 2 nM for thermolysin and elastase, respectively). To confirm the role of hydroxamate group in the inhibitory mechanism, similarly to the previous paper, hydroxamic acid moiety was replaced with carboxylate and hydrazide. The *K*_i_ values for all of the investigated enzymes increased from the nM to μM–mM range. Altogether, the configurational difference between **6A** and **6B**, both with hydroxamate moiety, enabled to distinguish the preferential interactions between human and bacterial enzymes.

Another good example of hydroxamates as collagenases inhibitors was presented in 1995 by Grams et al. [65]. They described X-ray structures of complexes between two peptide sequences and collagenase. This research was a follow-up of their previous work, in which the X-ray structure of collagenase with Pro-Leu-Gly-NHOH was examined [66]. Their observations led to the synthesis and evaluation of thiol inhibitor HSCH_2_CH(CH_2_Ph)CO-L-Ala-Gly-NH_2_ and hydroxamate inhibitor HONCOCH(*i*Bu)CO-L-Ala-Gly-NH_2_. Both functional groups bind to the catalytic zinc ion, however the hydroxamate inhibitor binds differently from the geometrical preferences revealed by the substrate. The calculated *K*_i_ values are: 19 μM for Pro-Leu-Gly-NHOH; 1.2 μM for thiol-modified sequence; 33 μM for hydroxamate peptidomimetic.

**Figure 3:**
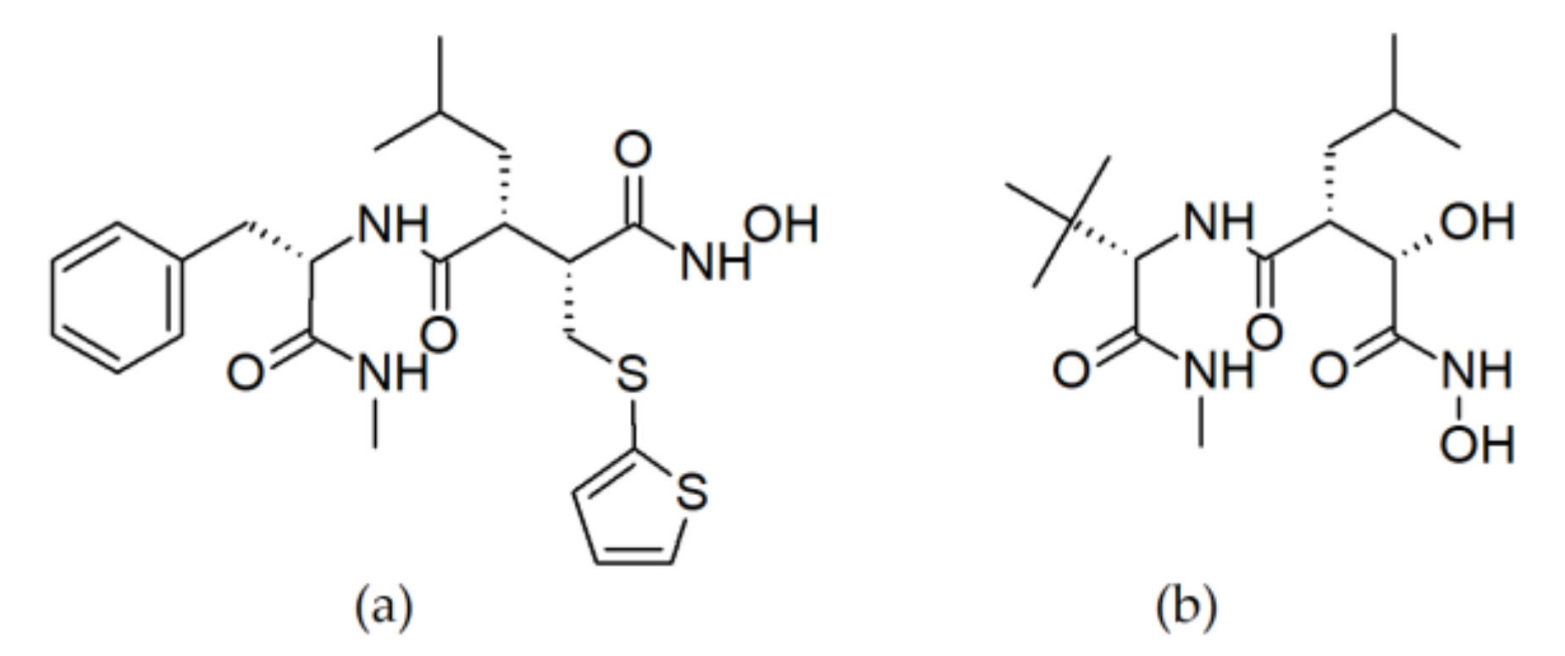
Chemical structures of: (**a**) batimastat; (**b**) marimastat.

Good examples of widely studied hydroxamates, batimastat (BB-94) and marimastat (BB-2516) (Figure 3), have to be mentioned in this section. Both molecules were approved as drugs after extended clinical trials, with relevant indications mostly in cancer therapies. Rasmussen et al. have published a very interesting review comparing the two substances and summarizing clinical data [67].

Batimastat binds covalently to Zn(II) ion in the MMPs active site. It exhibits a satisfactory influence on various MMPs, e.g., MMP-1, 3, 2, 9, and 7 (with IC_50_ values equal to 3, 50, 4, 4 and 6 nM, respectively). Mimicking the substrate of metalloproteases, acts as competitive and reversible inhibitor. For all advantages of batimastat, it has one weakness significantly diminishing its applicability—due to its poor solubility in water, has a very-low bioavailability after oral administration. On the contrary, marimastat can be administered orally with adequate results. It binds both to the zinc(II) ion and the binding site of the enzyme. Marimastat is almost as potent as batimastat, revealing reduced activity only against MMP-3 (IC_50_ values of 5, 6, 230, 16 and 3 nM against MMP-1, 2, 3, 7, and 9, respectively). New MMP-13 inhibitors based on hydroxamates were developed by Gooljarsingh et al. in 2007 [68]. Pyrimidine dicarboxamide, hydroxamate mimic, and acetohydroxamate were evaluated in the context of MMP-13 inhibition, resulting with nanomolar IC_50_ values for pyrimidine dicarboxamide and hydroxamate mimic, and millimolar values for the less potent acetohydroxamate.

In conclusion, hydroxamate-modified peptide inhibitors have been known for years, but they have been less studied in the last two decades. However, they are undoubtedly a group of compounds which played a pioneering role in collagenases inhibitors studies and that, in our opinion, could still offer pertinent starting points for the development of active ingredients relevant to the cosmeceutical sector.

#### 3.1.2. Phosphinic acid Derivatives

**Figure 4:**
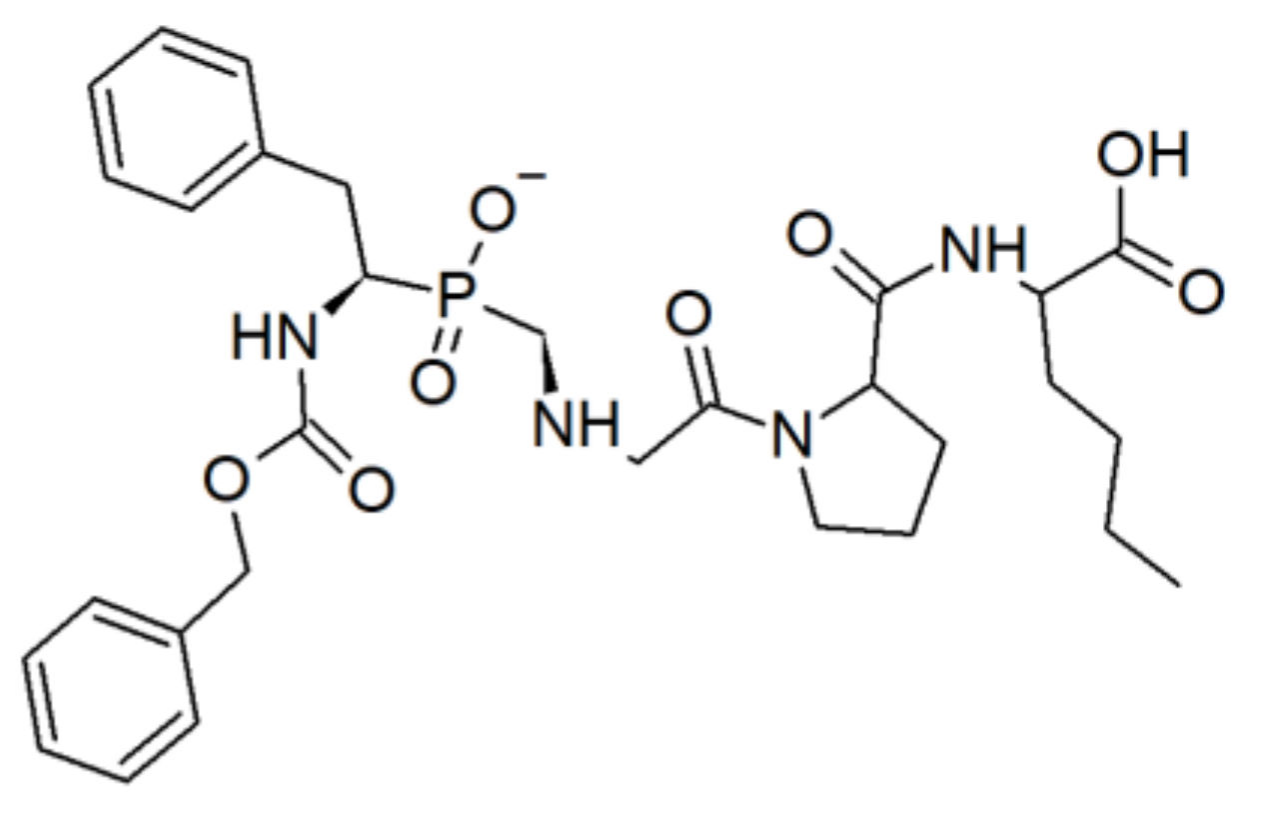
Phosphinic peptide Z-Phe-*ψ*(PO_2_CH_2_)-Gly-Pro-Nle, a potent inhibitor of collagenase, reported by Yiotakis et al. [69].

Shortly after the discovery of hydroxamate peptides acting as collagenases inhibitors, several compounds bearing the phosphinic acid moiety have been reported. In 1994 Yiotakis published two papers describing linear and cyclic peptides containing phosphinic group (PO^2−^), relevant for bacterial collagenases inhibition [69,70]. Substrate analogues of *Corynebacterium rathayii* collagenase were synthesised, replacing the scissile peptide bond by phosphinic moiety. Tetrapeptide Z-Phe-*ψ*(PO_2_CH_2_)-Gly-Pro-Nle (Figure 4) exhibited the collagenase inhibition with *K*_i_ value of 8 nM. Then, the influence of chain elongation was investigated. It was proven that the heptapeptide Z-Phe-Gly-Pro-Phe-*ψ*(PO_2_CH_2_)-Gly-Pro-Nle-OMe is an even more effective inhibitor than his shorter analogue (*K*_i_ = 0.6 nM). Further modifications of the tetrapeptide, including CH_2_ replacement by NH group, demonstrated insignificant decrease of *K*_i_ value from 8 nM to 6 nM.

Cyclic analogues of above-mentioned sequences were studied in tandem [70]. Generally speaking, the importance of conformationally constrained peptides was underpinned and investigated for various applications, mainly for drug-design purposes [71,72,73,74,75]. The design of new cyclic collagenase inhibitors was based on the following rules: (1) at least four interacting residues should be present; (2) there should be a bulky, hydrophobic group present in the P_1_ position; (3) it is required to incorporate glycine and proline in P_1’_ and P_2’_ positions, respectively; (4) P_3’_ position should be occupied by either linear, basic or apolar side chain residue (lysine or aminocaproic acid, Ahx). Cyclo[Gly-Pro-Phe-*ψ*(PO_2_CH_2_)-Gly-Pro-Ahx] together with cyclo[βAla-Pro-Phe-*ψ*(PO_2_CH_2_)-Gly-Pro-Ahx] were found to be relatively efficient, but not excellent inhibitors of this bacterial collagenase (*K*_i_ = 120 nM and 90 nM, respectively), comparing to the linear analogue Z-Phe-*ψ*(PO_2_CH_2_)-Gly-Pro-Nle (*K*_i_ = 8 nM). The role of configuration L and D was studied additionally, putting in a clear evidence that L analogues are favourable. Therefore, it can be summarized that, linear peptides in L conformation, fulfilling the above-mentioned requirements, are better candidates for collagenase inhibitors; nevertheless, also cyclic structures are promising.

**Figure 5:**
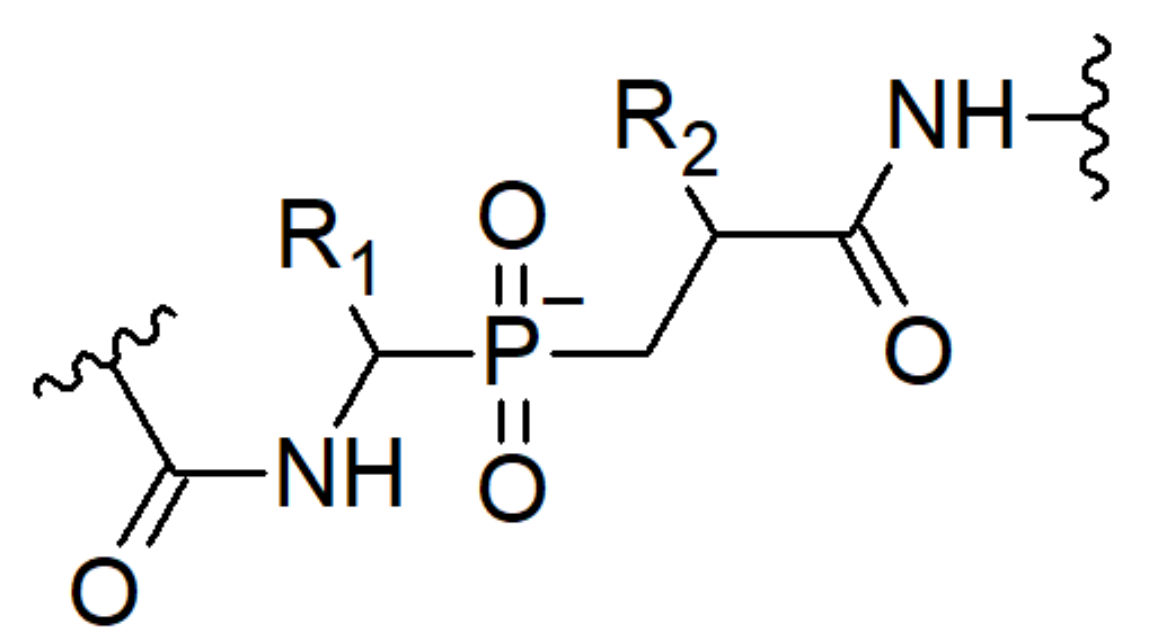
Common structural motif in phosphinopeptides [76].

Phosphinic pseudodipeptides (PPDs) were described by Bhowmick, Fields et al. in 2011 and 2012 [76,77]. All examined compounds were synthesised as building blocks in the Fmoc-containing form for further peptidomimetic development, with potential application as MMPs inhibitors. In 2011, Fmoc-protected phosphinic analogues of Gly-Val and Gly-Leu were obtained, but supplemental inhibition assays were not performed. Additionally, in 2012, another PPD Fmoc-Gly-Ile was successfully developed. All derivatives structures were based on common phosphonic peptides arrangement, reported in Figure 5.

Dive group achievements together with other scientists accomplishments in the field of phosphinic MMPs inhibitors, were summarized in the review by Yiotakis et al. in 2004 [78]. Furthermore, it was reported by Gall et al. that the phosphinic moiety incorporation led to a good inhibition of stromelysin-3 (MMP-11) as well [79]. Moreover, Lauer-Fields et al. proven a relevance of phosphinic peptides, a triple-helical transition state analogues, in MMP-2 and MMP-9 inhibition [80].

#### 3.1.3. Unmodified Peptides

Whilst hydroxamate and phosphonic modifications represent the main direction of collagenase inhibitors design about 30 years ago, after 2000 scientists begun to examine also unmodified sequences. Mostly, these peptides derive from proteolytic digestion fragments of other proteins from natural sources, mimicking biological substrates. Recognized by the collagenase, these peptides occupy its active site and decrease the catalytic ability. Another pathway comprises indirect activity when the aforesaid fragments are acting as signal peptides. Herein we will report sequences described within past 20 years, possessing effective collagenases inhibitory properties.

##### Synthetic Peptides

Oono et al. in 2002 investigated an influence of human neutrophil peptide-1 (HNP-1) on MMP-1 and proα1-collagen type I expression [81]. However in this paper the HNP-1 sequence was not reported, while it can be found online as commercially available product: H-ACYCRIPACIAGERRYGTCIYQGRLWAFCC-OH (**A**) [82]. To explore the possibility of HNP-1 application in wound healing, these authors used one-step reverse transcription polymerase chain reaction (RT-PCR) for semiquantitative analysis of mRNA of MMP-1 and proα1 collagen (type I and III). ELISA test was then applied in order to quantify the production of MMP-1 by fibroblasts treated and not treated with HNP-1. Their experiments suggested that HNP-1 upregulates collagen synthesis and decrease collagenase activity.

Field’s research group investigations have shown a significant role of peptides as inhibitors of MMPs, not only collagenases [83]. Among others, MMP-1 activity was noticeably decreased in the presence of STX-S4-CT peptide (sequence **C**) [84]. *K*_i_ value was calculated for 4.5 μM. Regasepin1, a short peptide **D**, was found to be a good and selective inhibitor of collagenases—IC_50_ value for MMP-8 (collagenase-2) was 3 μM; in the case of MMP-1 and MMP-13 (collagenases-1 and -3) this value increased up to 100 μM [85]. Finally, TM8 peptide **E** inhibited all three collagenases well, with the *K*_i_ values of 10, 28 and 12 nM for MMP-1, -8, and -13, respectively [86].

**Figure 6:**
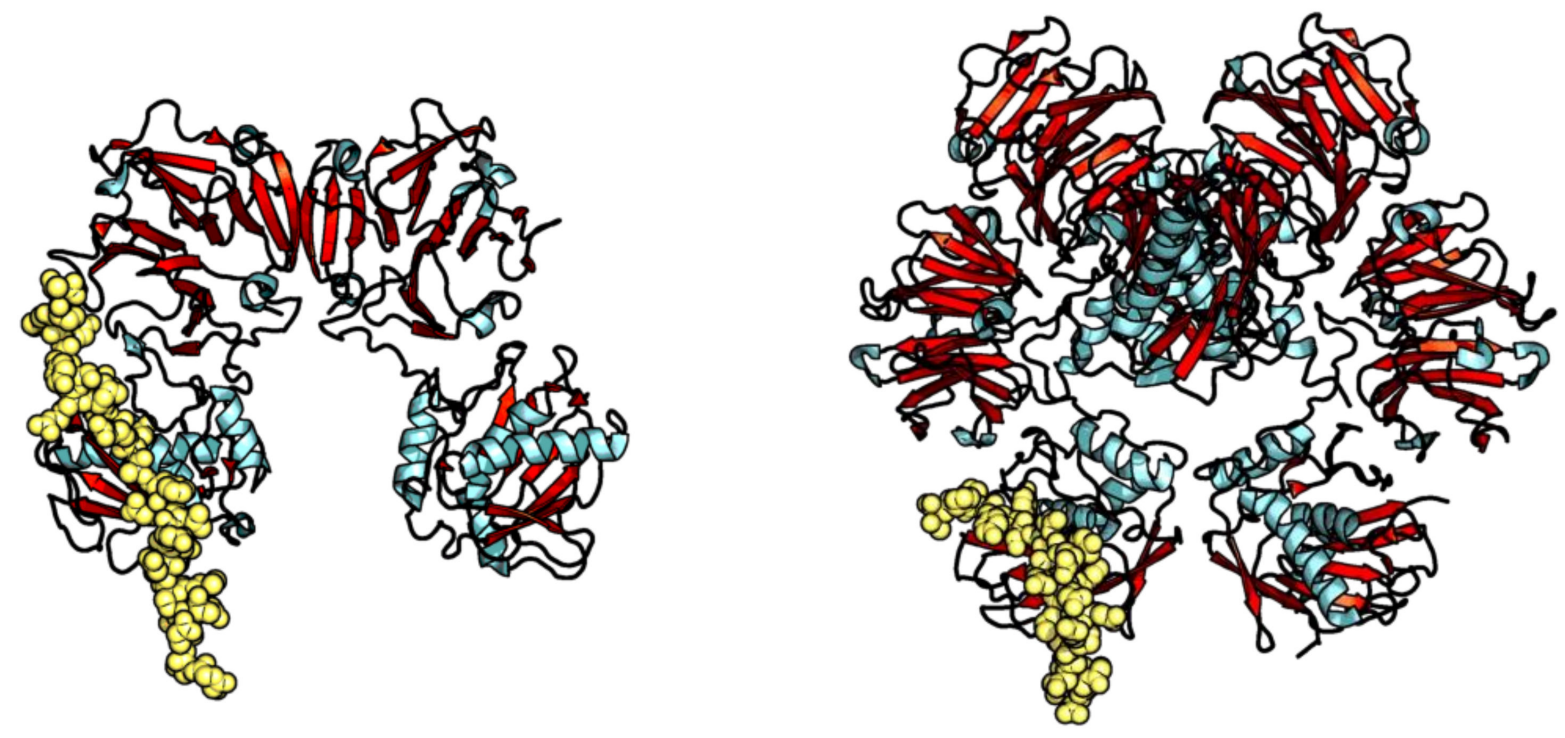
Human collagenase 3 (MMP-13) in two complexes with peptide **F** (PDB codes: 4FU4, 4G0D) [87]. The inhibitor molecules are presented as spheres. All figures of crystal structures were prepared in PyMOL [88].

**Figure 7:**
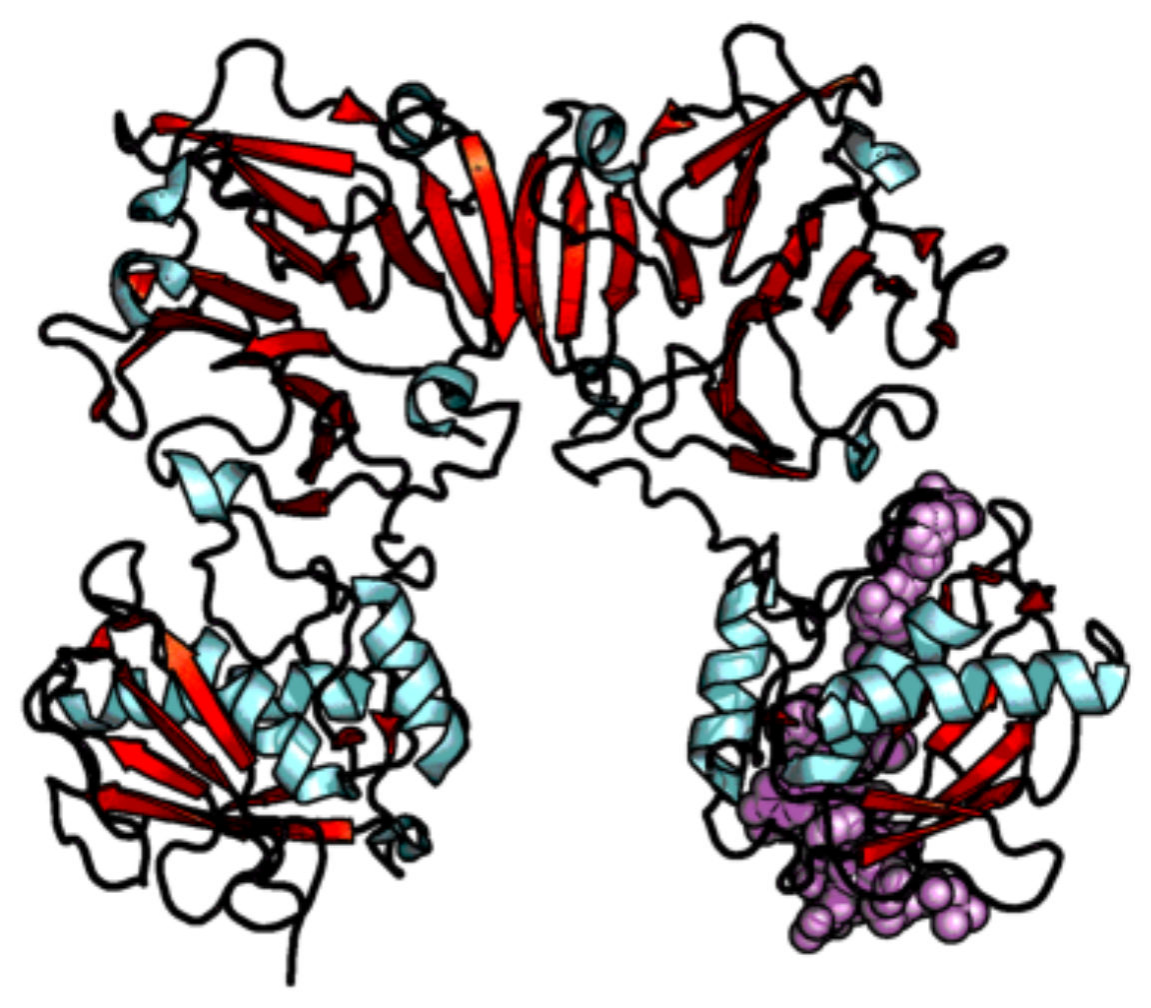
Human collagenase 3 (MMP-13) in the complex with peptide **G** (PDB code: 4FVL) [87].

The crystallized complexes of catalytically inactive human collagenase-3 (MMP-13) with peptides were described by Stura et al. in 2013 [87]. Two different peptides (**F** and **G**) deriving from the cleaved prodomain proMMP-13 throughout activation were bound to the enzyme active site. N-terminal modifications within these sequences were variable and showed interactions in different parts of catalytic domain. Structural details of crystals with relatively long peptide chains (up to 26 residues) can be found in Protein Data Bank library (codes 4FU4, 4FVL, and 4G0D). They refer to the complexes with the following peptides: G^25^GDEDDLSEEDLQFAERYLRSYYHPT^50^ (Figure 6, left), D^30^LSEEDLQFAERYLRSYYHPT^50^ (Figure 7), and G^25^GDEDDLSEEDLQFAERYLRSYYHPT^50^ with the full enzyme (Figure 6, right), respectively. This study does not depict typical inhibitory properties of the mentioned sequences. Nevertheless, it can be a good starting point for new compounds development due to the structural properties and interactions found in crystals.

The relevance of MMP-13 inhibitors as a useful tool for decreasing the collagen degradation was confirmed by another study of Howes et al. [89]. Their investigations have shown that MMP-1 acts differently to MMP-13 and they recognize diverse collagen cleavage sites. Moreover, MMP-13 digest preferably collagen type II over types I and III, cleaving the α chains sequentially. To check the recognition sites in collagen, these authors synthesised a library of overlapping homotrimeric peptides, including a 27 amino acids sequence from primary collagen and repeatable (GPP)_5_ fragments (located both on N- and C-terminus), providing a triple-helical conformation. Their studies revealed the highest affinity of MMP-13 to two sequences, so-called II-44 and II-8 (**H** and **I**, respectively). The first peptide, II-44, was found to occur in a triple helix form in 80%; for II-4 this value was 48% (at 37 °C). Calculated IC_50_ for both compounds shown a higher inhibitory potency for II -44 (IC_50_ = 8 μg/mL), while II-8 was a fourfold weaker inhibitor of MMP-13. Moreover, it was confirmed that the fragments essential for the recognition and responsible for a robust binding are GL*X*GQR, found twice in II-44 and not found in II-8. Therefore, it was possible to explain the slight difference observed between the obtained results.

Fields research group, previously mentioned, investigated MMP-13 triple-helical inhibitors **J**, **K**, and **L,** obtained using *click* chemistry [90]. These authors found that assembling of three different chains into heterotrimeric helices provides adequate thermal stability and results in MMP-13 and MT1-MMP inhibition in the range 100–400 nM. What is more, the heterotrimeric analogues were selective between MT1-MMP and MMP-1, on the contrary to homotrimer-based structures, favouring MMP-13.

##### Peptides from Natural Sources

In 2011, Park et al. published an article delineating the isolation of a peptide inhibiting MMP-1, derived from *Dipturus chilensis* skate skin protein [91]. The extracted protein was then digested by various enzymes, e.g., alcalase, α-chymotrypsin, trypsin, neutrase, papain, and pepsin. Obtained cocktails were then tested against collagenase-1, exhibiting an inhibitory effect of pepsin hydrolysate. Bioactive peptide was purified, and mass spectrometry confirmed the molecular weight value 961 Da of purified product **B** Additionally, the IC_50_ value for the isolated peptide was 87.0 μM.

#### 3.1.4. Amino Acids and Peptide Derivatives

**Figure 8:**
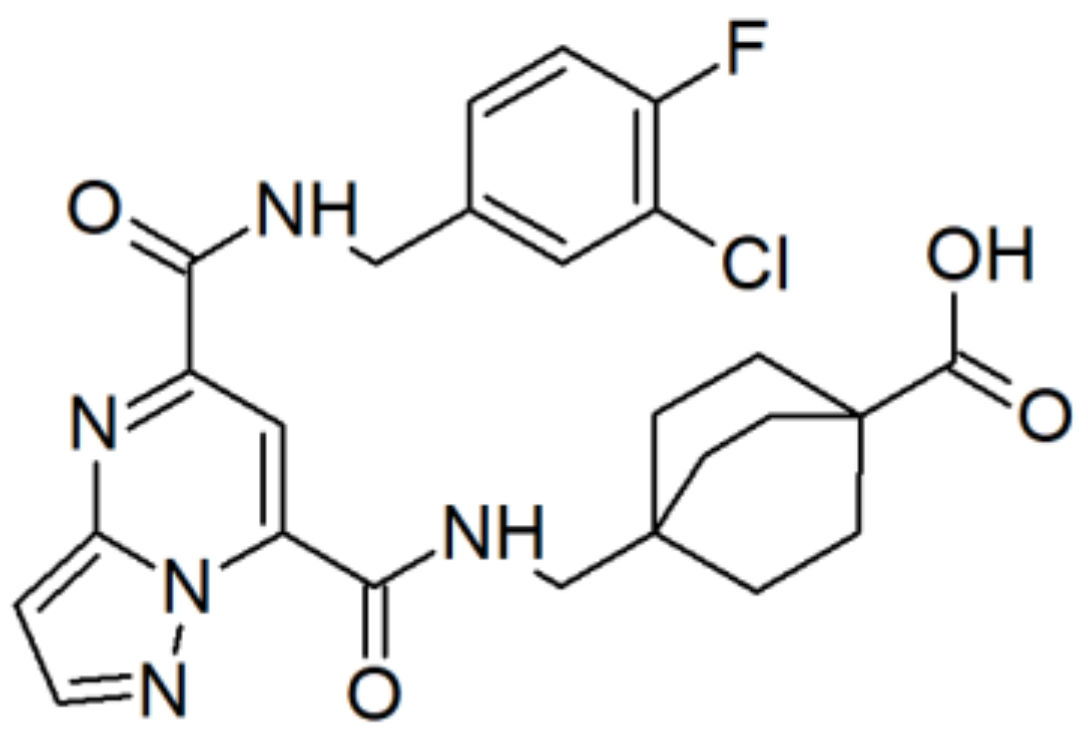
Chemical structure of MMP-13 inhibitor (MMP13i-A) [92].

Apart the above-mentioned categories of collagenases inhibitors, there are other uncategorizable molecules. One of them is MMP-13 inhibitor (MMP13i-A) described in 2011 by Quillard and co-authors [92]. It is a nonhydroxamate molecule, significantly decreasing MMP-13 activity (Figure 8). What is particularly interesting, among all tested MMPs (1, 2, 7, 8, 9, 12, 13, and 14) only MMP-13 was efficiently inhibited (IC_50_ = 33.5 nM). Other metalloproteases shown no inhibition with IC_50_ value over 20000 nM. These observations suggested a certain selectivity and were applied during in vivo observations, including macrophage accumulation and MMP-13 activity monitoring. Mice treated for 10 weeks with MMP-13i-A, revealed reduced activity of MMP-13 without affecting macrophage content, measured by molecular imaging.

A great example of cosmeceutically relevant compounds are Mycosporine-like Amino Acids (MAA) from marine sources [93]. According to the paper by Hartmann et al., three bioactive molecules were firstly isolated from the marine red algae (*Porphyra sp.*, *Palmaria palmata*) and then chromatographically purified for further use. Inhibitory properties of shinorine, porphyra, and palythine were evaluated against *Chlostridium histolyticum* collagenase. Obtained IC_50_ values are presented in the Table 3. To compare, two standard inhibitors phosphoramidon and 1,10-phenanthroline were considered additionally in the assay. Furthermore, molecular docking study confirmed the competitive mechanism of inhibition revealed by tested MAAs, with IC_50_ values between 100–160 μM.

In two papers from Fields, Roush et al., mentioned in the introduction of collagenases section, the authors evaluated inhibitory activity of various amino acids derivatives against MMP-13 [61,62]. They reported the two potent and selective inhibitors, **10d** and **(*S*)-17b**, occupying the MMP-13 binding site and surrounding the catalytically active Zn^2+^ ion, without its chelation. IC_50s_ found for these two derivatives were calculated as 3.4 nM and 2.7 nM, respectively. Then, Fuerst et al. identified the two compounds **2** and **31**, exhibiting improved microsomal half-life, kinetic solubility, and permeability coefficient. Both compounds were selective among three tested MMPs (IC_50_ values for compound **2**: 2.7 nM for MMP-13 and over 5000 nM for MMP-2, MMP-8; for compound **31**: 8.5 nM, over 5000 nM, and 832 nM for MMP-13, -2, and -8, respectively).

A wide and detailed review describing inhibitors of MMPs in general, including collagenases, was published in 2020 by Raeeszadeh-Sarmazdeh et al. [94].

### 3.2. Elastase

In 1949 Baló and Banga published a communication in Nature, claiming that fresh pancreas extraction as well as dried pancreas powder “contains a specific enzyme, called ‘elastase’” [95]. Thus, this was the first scientific statement describing this important enzyme, which was thereafter investigated by other researchers. Tsuji and co -workers delineated the role of fibroblast elastase in wrinkle formation, providing also some indications in their inhibitors design [96]. These authors proven the importance of skin fibroblast elastase in elastic fibre degradation. At this point, it is worthy to underline that at least two elastase types are present in the skin, i.e., neutrophil and skin fibroblast elastase. While the first is a serin protease, the latter belongs to the metalloprotease family [97]. Because both of them have the ability to degrade fibrillar collagen, we will highlight the interesting inhibitors of each one.

In a paper by Imokawa group, the authors evidenced an undoubted difference between these elastases [96]. Comparing different enzyme inhibitors, inhibiting profiles for both neutrophil and fibroblast elastase were created. Among PMSF (phenylmethyl sulfonylfluoride), elastatinal, leupeptin, pepstatin A, EDTA, 1,10-phenantrolin, and phosphoramidon, the last one (already listed in Table 2) together with EDTA and, 1,10-phenantrolin (all known as metalloprotease inhibitor), significantly decreased the fibroblast elastase activity. On the other hand, neutrophil elastase remained active in these conditions, inhibited only by PMSF and elastatinal (typical serine protease inhibitors). Thus, the dissimilarity between the mechanisms of action of the two elastases was proven and explained. Additionally, the work by Azmi et al. showed a relevant inhibitory activity of elastase, tyrosinase, and MMP-1 by worm extracts, indicating its potential application in the cosmeceutical field [98]. However, due to the biological extract incorporation instead of purified compounds, precise inhibitory differences among three evaluated enzymes cannot be clearly distinguished.

For more detailed papers about biological activity and functions of human neutrophil elastase (HNE), together with examples of diverse inhibitors, we indicate a set of review papers by Ohbayashi et al., Kelly et al., Groutas et al., and a recently released one by Ahmad et al. [99,100,101,102].

#### 3.2.1. Chloromethyl Ketones

One of the very first papers describing the inhibition of porcine pancreas elastase (PPE), human leukocyte elastase (HLE), and cathepsin G (CatG) was published in 1977 by Powers et al. [103]. The cleavage pattern of various peptides by PPE was examined, thus determining substrate structural preferences. Afterwards, a few new chloromethyl ketones Ac-Ala-Ala-Pro-AACH_2_Cl (where AA = Ile, Val, Thr) were synthesised. PPE was inhibited more rapidly by the peptide bearing Ile, while the Thr variant did not show significant inhibition. On the other hand, HLE was inhibited more efficiently by sequences including Ile and Val. MeO-Suc-Ala-Ala-Pro-ValCH_2_Cl, soluble in water, was established as a very-good inhibitor of HLE; Z-GIy-Leu-PheCH_2_Cl was effective against cathepsin G, but not leukocyte elastase.

**Figure 9:**
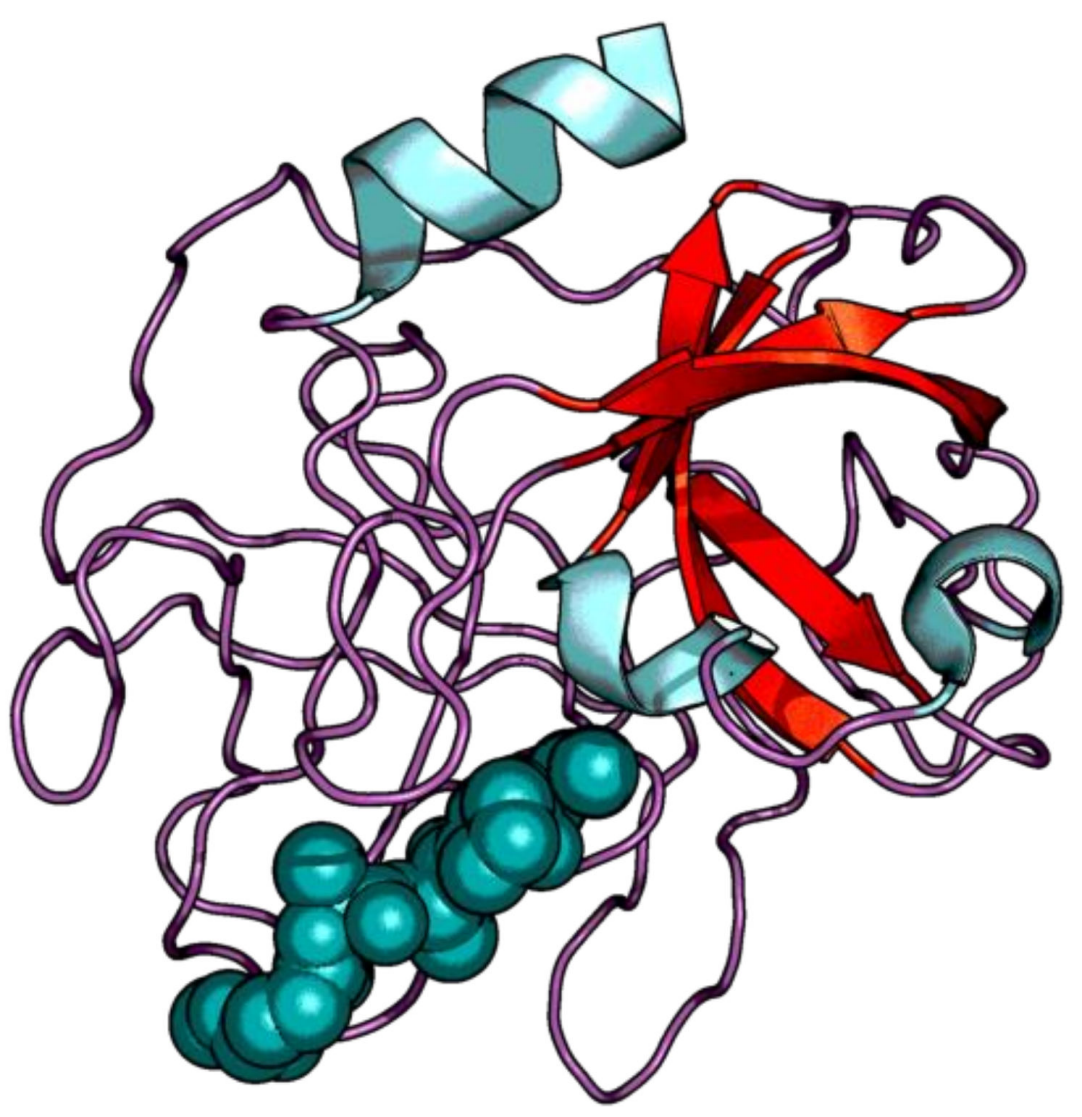
Structure of human neutrophil elastase in complex with MSACK (PDB code: 1HNE) [104]. The inhibitor molecule is presented as spheres.

In 1989, Navia and co-workers presented a work in the field of peptide chloromethyl ketones as HNE inhibitors [104]. They have noticed some side effects of peptides evaluated by Powers et al. in humans, and therefore designed new molecules with diminished toxicity. Crystal structure of methoxysuccinyl-Ala-Ala-Pro-Ala chloromethyl ketone (MSACK) is presented in Figure 9. It was proven that MSACK is able to form the cross-linkage between the His-57 and Ser-195 residues, responsible for the catalytic activity.

In vivo experiments on rats were reported in the paper by Cowan et al. [105]. Animals with advanced pulmonary vascular disease were then treated with peptidyl trifluoromethylketone serine elastase inhibitors, so-called ZD0892 and M249314. It was demonstrated that untreated rats have shown 900% loss of cellular matrix after 21 days, with further increase of 800% within next week. After M249314 administration, elastase inhibitory activity was noticeable over the first 6 hours, and then decreased but remained evident until the second dose. What is more, the mortality of sick and untreated rats has begun after 23 days, while after 28 days only one rat from each group (ZD0892 and M249314-treated for 1 week) was died. Summarizing, the survival of M249314-treated rats was 86%, whereas all untreated rats had died by 30 days of the trial.

#### 3.2.2. Cyclic Depsipeptides 

**Figure 10:**
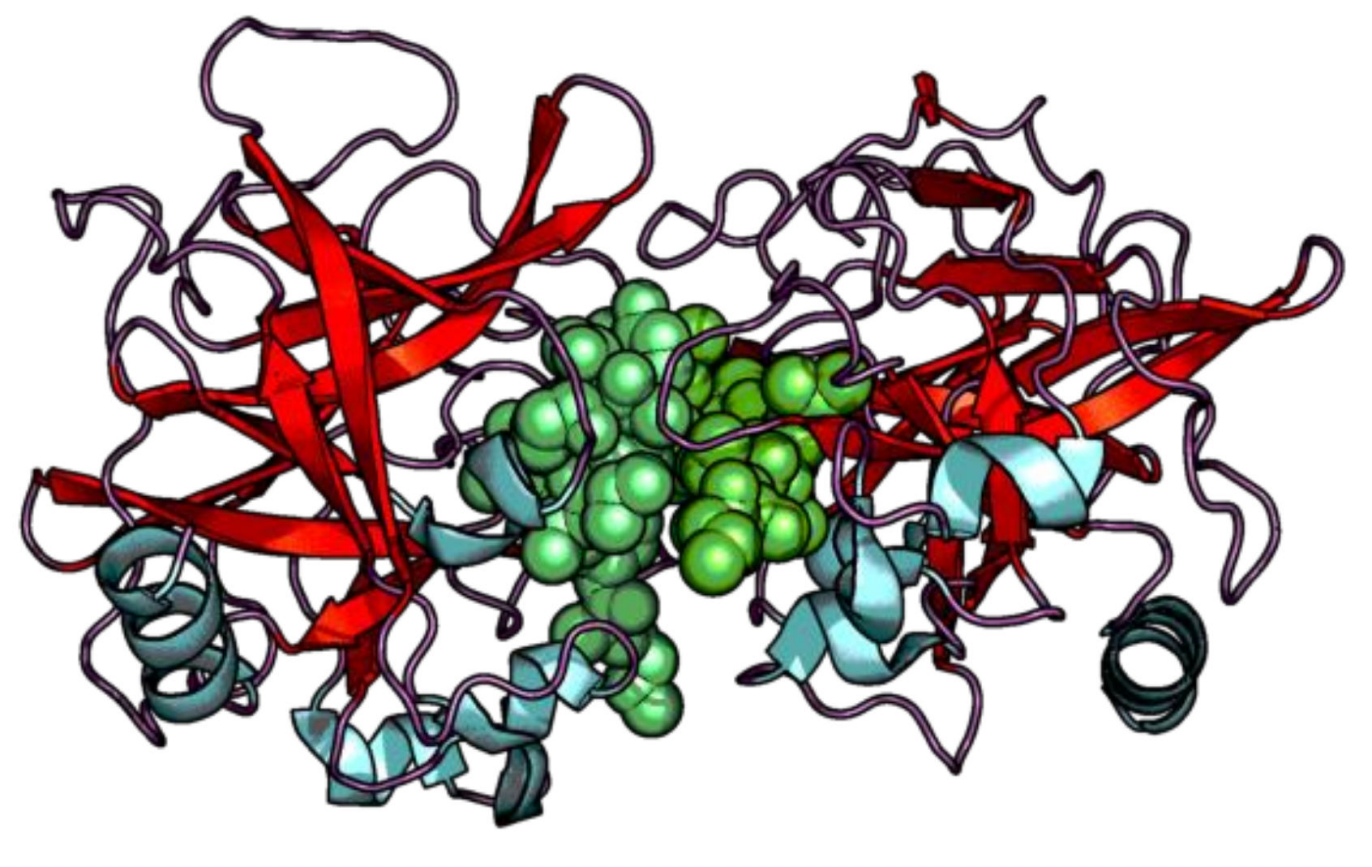
Binding structure of elastase inhibitor Scyptolin A (PDB code: 1OKX) [107]. The inhibitor molecules are presented as spheres.

Advantages of cyclic structures were already mentioned previously in the paragraph related to collagenases. In 2001 and 2003, Matern et al. described the inhibition of PPE by scyptolin A and B, together with the crystal structure of scyptolin A bound to this enzyme [106,107]. Two cyclic depsipeptides (peptides with one or more amides replaced by the ester groups) were isolated from cyanobacterium *Scytonema hofmanni* PCC7110, namely scyptolin A and B. Both molecules were selective inhibitors of PPE, exhibiting IC_50_ values of approximately 3 mg/mL. The crystal structure has shown the rigid ring of scyptolin A occupying the elastase active site, thus preventing a hydrolytic attack and decreasing the enzyme activity (Figure 10). Due to the similarity of PPE and human elastase, the structures of scyptolin A and B appear as valuable starting points for further investigations in the field of human elastase inhibitors.

Another interesting example of cyclopeptides from natural sources, relevant as elastase inhibitors, were presented a recent paper by Keller et al. [108]. These authors described three new cyclic peptides, so-called tutuilamides A–C, structurally defined by various techniques such as NMR and chemical derivatization. Particularly, 3-amino-6-hydroxy-2-piperidone and 2-amino-2-butenoic acid, and a novel vinyl chloride residue, were found in these sequences. IC_50_ values obtained during the PPE inhibition assay were 1.18, 2.05 and 4.93 nM for tutuilamide A, B, and C, respectively. Moreover, trypsin inhibition was not found in the presence of the discussed cyclopeptides (IC_50_ > 20 000). X-ray crystallography revealed the formation of hydrogen bond between the amino acid backbone of tutuilamide A and the enzyme binding pocket, thus stabilizing the complex end explaining the inhibitory activity.

#### 3.2.3. Unmodified Peptides

Structurally unmodified sequences can be found in the literature, too. In 1995, Toth et al. published the results of in vitro and in vivo studies, concerning lipophilic peptide inhibitors of human neutrophil elastase [109]. Four lipopeptides, namely **1b**–**e**, shown relevant HNE inhibition. Obtained IC_50_ values were 2.9 × 10^−9^, 2.8 × 10^−10^, 1.8 × 10^−l0^, and 4.1 × 10^−l0^ mmol × mL^−1^, for **1b**, **1c**, **1d**, and **1e**, respectively. It was found that the inhibition was increasing while the lipophilicity of sequences was higher: moreover, carboxylic acid esterification in analogue **1e** led to decreased inhibitory effect. All sequences were based on X-AAPV-Y fragment, with proper N- and C-terminal modifications (X and Y, respectively). All evaluated sequences are reported in the Table 4. In vivo experiments on rabbit skin proved the protection of elastic fibre against elastolysis, exhibited by the lipopeptide **1d** (**M**) administered intradermally. 

In 2000, Hilpert et al. designed and conducted a very broad investigation in order to identify and finally modify a sequence for efficient PPE inhibition [110]. Shorter chains were designed on the basis of OMTKY3 (third domain of turkey ovomucoid inhibitor), exhibiting the *K*_i_ value of 5.5 × 10^−12^ M. Synthesis of peptides together with their cyclisation were performed on cellulose membranes, and were followed by the binding affinity measurements carried out on the membrane, too. The significance of each amino acid was determined by the exchange of every residue with each one of the other 19 coded amino acids. Thereby, over 700 different sequences were evaluated in binding to PPE, and 320 of them were then measured in the inhibition assay in microtiter plates with punched out peptide spots. Interestingly, cyclization did not provide any beneficial interactions in the active cleft and did not provoke an extended inhibition. Among all tested sequences, the shortest one exhibiting the 84% decrease of PPE activity, was peptide **N**. *K*_i_ values were of 6.7 × 10^−7^ M, 1.1 × 10^−4^ M, and 1.3 × 10^−4^ M for PPE, HLE, and BPC (bovine pancreas α-chymotrypsin), respectively. Furthermore, the described experiment including **N** sequence has been continued and broaden into the hybrid miniprotein design, inhibiting PPE likewise [111].

Another study on synthetic peptides as HNE inhibitors was performed by Vasconcelos et al. in 2011 [112]. Inhibitory effect on HNE and PPE of two sequences were compared to commercially available elastatinal. These sequences were based on Bowman–Birk protein-derived peptides able to inhibit HNE, summarized by McBride et al. [113]. Peptides **O** and **P** resulted to act as competitive inhibitors of HNE, with higher efficacy found for the sequence of peptide **P**. Probably, the increased hydrophobicity conferred by the (GA)_7_ tail provided relevant interactions with the enzyme, thus confirming the results reported in the previously mentioned work by Toth et al. [109]. Obtained IC_50_ values were at micromolar level (for the peptide **2**: 8.1 and 6.3 μM; for the peptide **P**: 7.0 and 0.4 μM in the case of PPE and HNE, respectively).

One more example of non-modified peptide sequence, applicable as elastase inhibitor, was presented in the paper by Wan et al. in 2013 [114]. Authors identified the first Kunitz-type serine protease inhibitor, namely AvKTI (**R**), found in *Araneus ventricosus* spider. The amino acid sequence was deduced from AvKTI cDNA from GenBank and established as KDRCLLPKVTGPCKASLTRYYYDKDTKACVEFIYGGCRGNRNNFKQKDECEKACTDH (57 residues). Its activity against plasmin and elastase was proven, with *K*_i_ values of 4.89 nM and 169.07 nM, respectively, whereas IC_50_ values were calculated as 10.07 and 446.93 nM, respectively.

#### 3.2.4. Peptide Derivatives and Cyclic Compounds

In this paragraph we would like to pay particular attention to the peptide and peptide-like sequences without functional groups belonging to the wider category described above, and exhibiting a relevant inhibitory activity of elastase. Therefore, macrocyclic compounds, together with unnatural amino acid-containing sequences will be described further. Exceptionally, we will also mention a few non-peptide molecules, in order to provide valuable indications for further design of new compounds.

**Figure 11:**
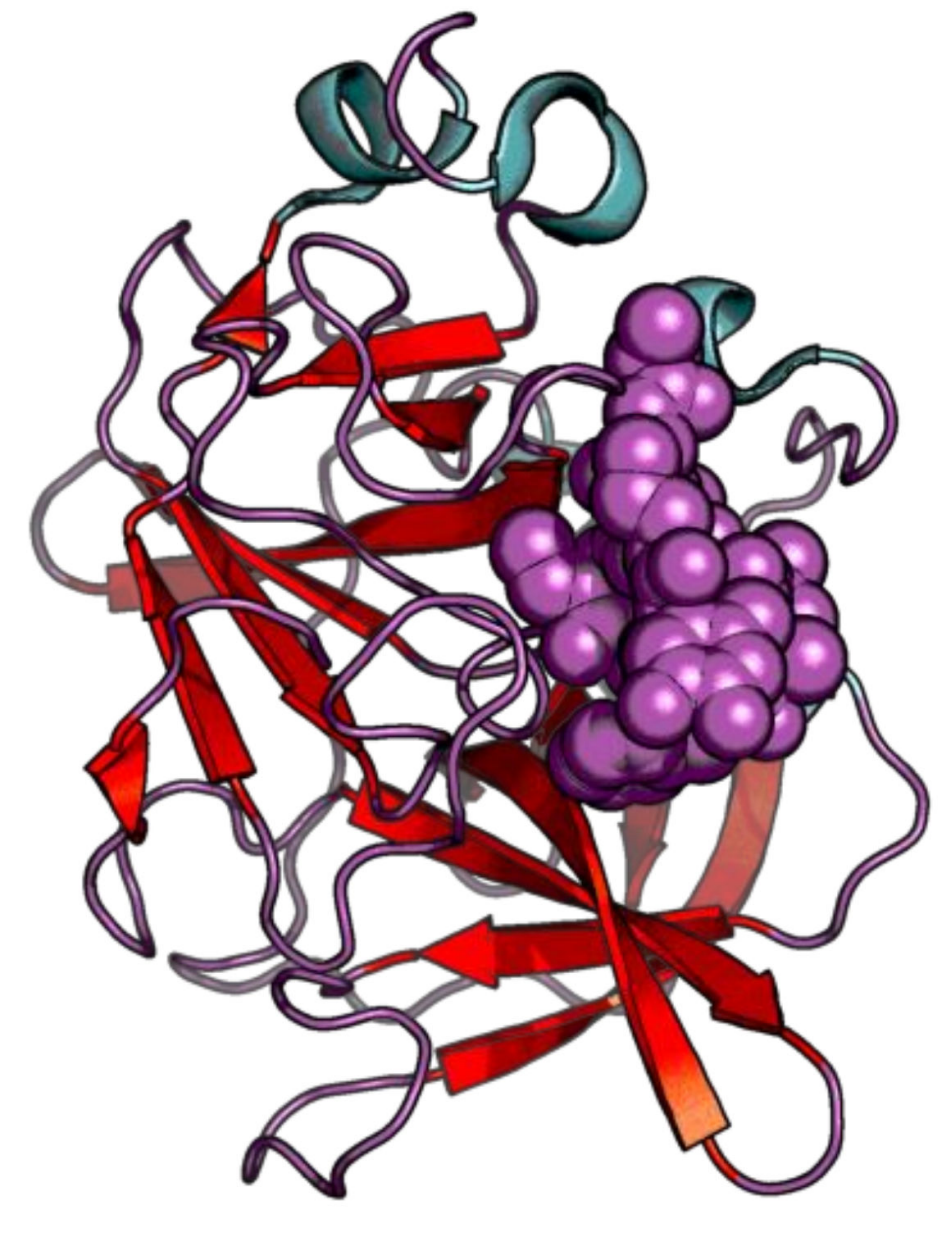
Crystal structure of porcine pancreatic elastase in complex with **S** (PDB code: 1QR3) [115]. The inhibitor molecule is presented as spheres.

**Figure 12:**
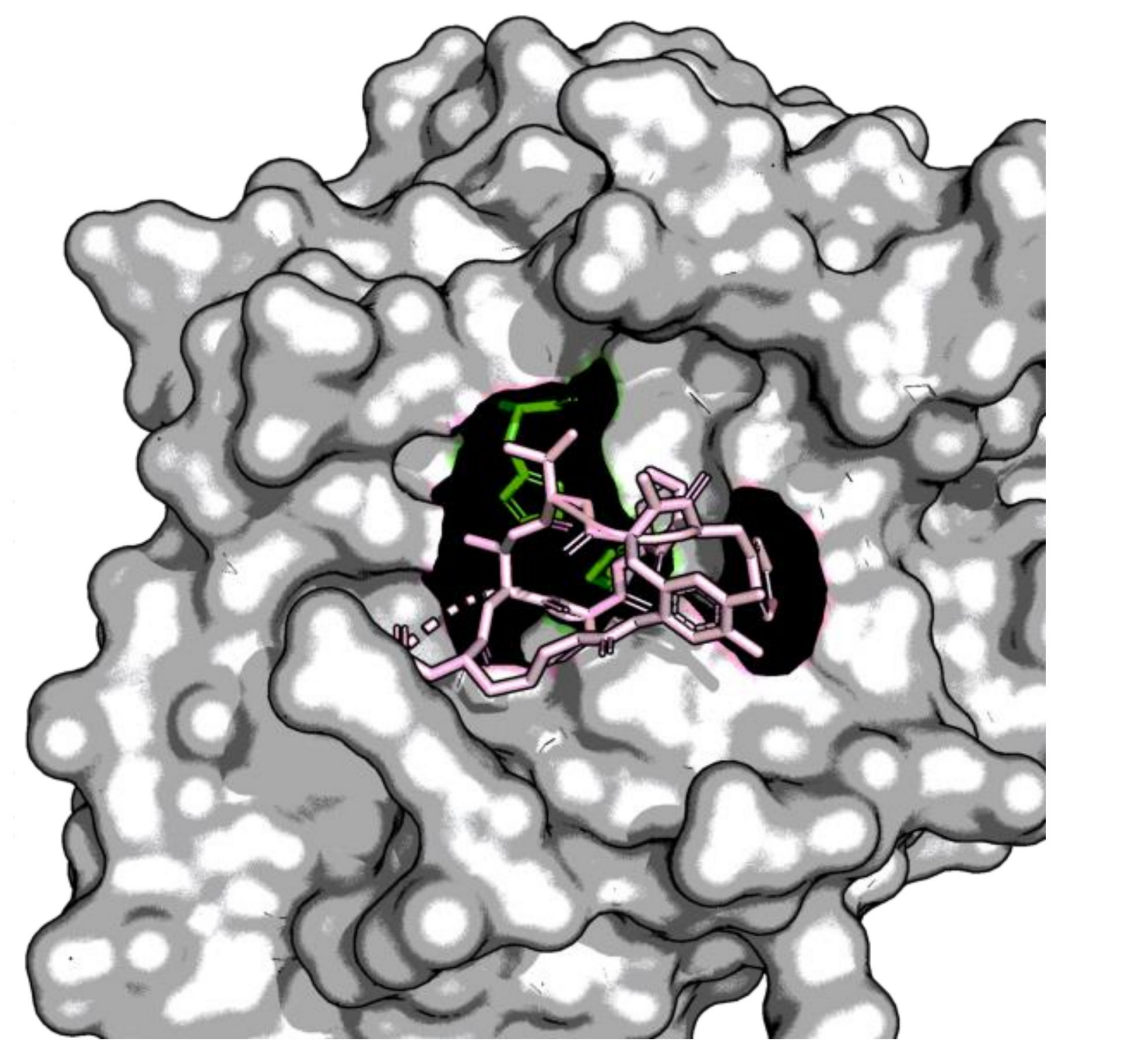
Crystal structure of porcine pancreatic elastase in complex with **S** (PDB code: 1QR3) [115]. The proximity of His-57 and Ser-195 residues (green) and the inhibitor molecule (pink) is visible.

In 2000, Nakanishi and co-workers determined the crystal structure of PPE in the complex with FR901277 inhibitor (**S**), a natural compound extracted from the culture filtrate of *Streptomyces resistomicificus* (Figure 11 and Figure 12) [115]. Previous examination of this compound against HLE and PPE, resulted with the IC_50_ value of 18 and 26 μM, respectively [116]. FR901277 consists of 7 amino acids, among which 4 are natural amino acids [L-Orn(1), L-Thr(2), L-Phe(5), and L-Val(7)] and 3 are unusual [dehydroxyThr(3), AA(4), and AA(6)], with the bridge between L-Thr(2) and L-Val(7). The crystal structure of PPE with FR901277 have shown the occupation of PPE binding subsites by inhibitor hydrophobic residues, with additional van der Waals contacts.

The relevance of elastase inhibition in cosmeceutical application has been already noticed in early 2000s by Tsukahara et al. [117]. These authors reported a significant role of elastase in the damage of dermal elastic fibres, leading to wrinkle formation. *N*-phenetylphosphonyl-leucyl-tryptophane (NPLT), an elastase inhibitor, was applied topically 5 times a week on rat skin, leading to diminished wrinkle formation and maintained skin elasticity. 0.1 ± 10.0 mM concentrations were tested, revealing the peak of efficacy at 1.0 mM concentration.

**Figure 13:**
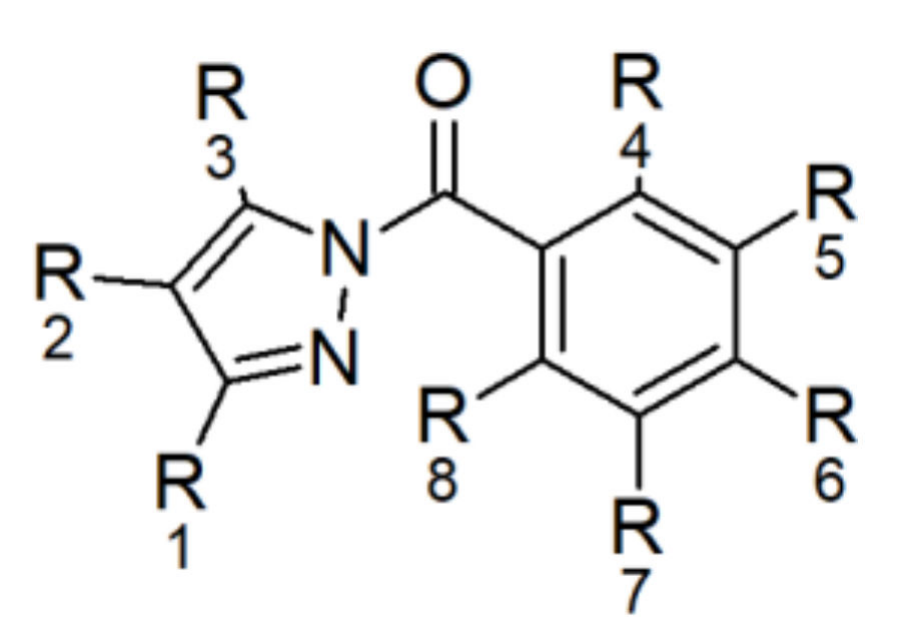
General chemical structure of *N*-benzoylpyrazoles.

Novel, low-molecular HNE inhibitors were described by Schepetkin et al. in 2007, setting a milestone in heterocyclic elastase inhibitors field [118]. Structures based on *N*-benzoylpyrazoles were eventually modified and optimized, leading to a library of potent compounds (for a general structure of *N*-benzoylpyrazoles, please see the Figure 13) and 17 molecules were then chosen for further experiments and SAR (structure-active relationship) analysis, revealing *K*_i_ values between 6-300 nM. Molecular docking studies were performed in order to explain the differences in potency among the most active and inactive compounds. It has been noticed that the carbonyl group of the inhibitor was located in the NE binding site, thus interacting with the catalytic triad. On the other hand, inactive analogues were either sterically hindered, or showed unpreferable orientation, hence excluding the presence of relevant interactions.

**Figure 14:**
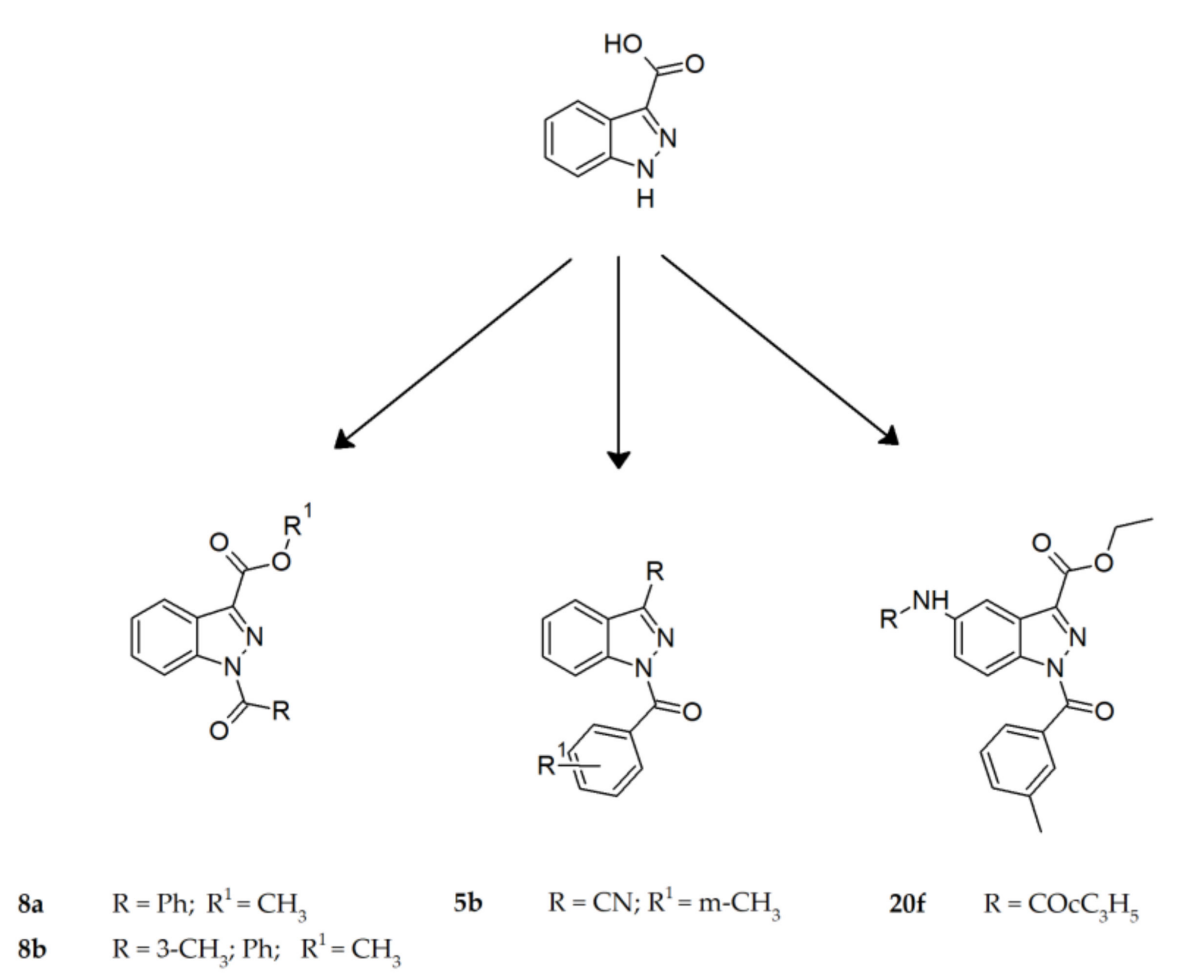
*N*-benzoylindazole derivatives presented in the papers by Crocetti et al. [119,120].

Starting from 2011, Giovannoni group was working on *N*-benzoylindazole derivatives acting as HNE inhibitors, progressing the work of above-mentioned Quinn’s investigations [119,120]. Among all tested compounds, so-called **8a** and **8b** showed the most potent, competitive and pseudo-irreversible inhibition, with IC_50_ values of ≈0.089 and ≈0.13 μM, respectively. Accordingly, also in this study molecular modeling have highlighted the importance of the carbonyl group in the *N*-benzoylindazole derivatives structures, due to the favourable interactions with -OH from Ser195 in the active cleft. Going forward, other analogues were developed to increase the inhibition efficacy and overall stability. Two more compounds, **5b** and **20f**, resulted with the inhibition level of IC_50_ ≈ 7 and 10 nM, respectively (Figure 14).

### 3.3. Cathepsins

Among a variety of cathepsins, the most relevant classes for cosmeceutical purposes are cathepsins K, L, and S (CatK, CatL, and CatS, respectively). These endopeptidases belongs to the cysteine cathepsins family and regulate several biological functions, such as inflammatory and proteolytic processes, or ECM remodeling [121]. Due to their role in fibrillar collagen degradation, a few examples of cathepsins inhibitors will be reported here.

Brömme and Lecaille summarized the features of the ideal CatK inhibitor, pointing to the importance of low molecular mass, minimal peptide character, reversibility and selectivity [122]. Aguda et al. evidenced that CatK is capable of fibrillar collagen degradation, underlining the participance of collagen-bound glycosaminoglycans in this process [123]. Additionally, a role of CatL in type I collagen degrading pathway was proved by Parks et al. [32]. It was found that CatL requires the presence of CatK to degrade effectively tendon ECM.

#### 3.3.1. Peptide Aldehydes

Peptidyl aldehydes as cathepsins inhibitors are known at least since 1969, when leupeptin (Ac-Leu-Leu-Arg-CHO, **T**) and antipain (N-[Nα-carbonyl-Arg-Val-Arg-al]-Phe, **U**) were described for the first time [124,125]. Then, in 1990 Sasaki et al. synthesised eight different di- and tripeptide aldehydes [126]. Each analogue contained L-leucine followed by an aldehyde located at the C-terminus (on L-norleucine, L-methionine, or L-phenylalanine). Performed enzymatic tests shown the inhibition of cathepsin B and L, with a particular efficacy against the second one. **V** inhibited CatL with *K*_i_ = 0.5 nM; **X** inhibited CatB with *K*_i_ = l00 nM.

Building on the efficacy of the above-mentioned analogues, Votta et al. in 1997 published a study dealing with other peptide-aldehyde inhibitors [127]. Two classes of compounds were distinguished, namely time-dependent and time-independent inhibitors. Cbz-Leu-Leu-Leu-CHO (**Y**) was selected to be the most potent one, classified into the time-independent group. The values of *K*_i_ and IC_50_ were 1.4 nM and 0.02 μM, respectively. Furthermore, **Y** reduced extensive bone loss provoked by CatK.

**Figure 15:**
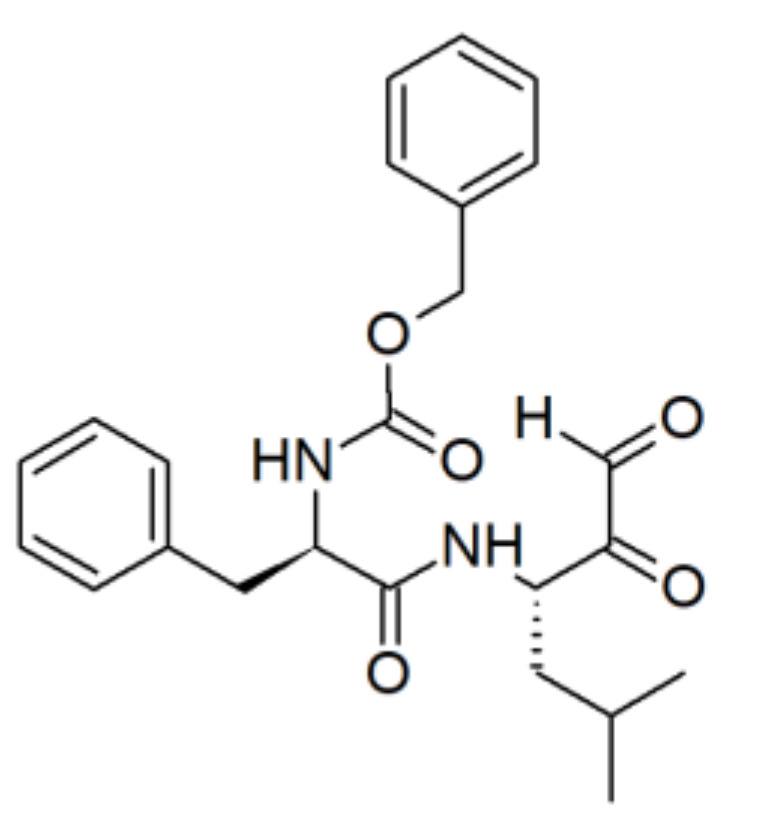
Cbz-Phe-Leu-COCHO (**Z**), one of the most relevant peptide aldehydes evaluated in the work by Walker et al., exhibiting CatS inhibition [128].

Other than CatK and CatL, also CatS became known to be inhibited by peptidic aldehydes. In 2000, Walker et al., from already mentioned Brömme group, published an evaluation of dipeptide α-keto-β-aldehydes (glyoxals) as new inhibitors of cathepsin S [128]. Through the solid-phase peptide synthesis (SPPS), a spectrum of dipeptide analogues was obtained and tested against cathepsin S. One of them, **Z**, exhibited good selectivity (*K*_i_ = 0.185 nM for CatS and 76 nM for CatB) (the structure can be found in the Figure 15).

#### 3.3.2. Peptidic Nitriles

Balicatib (AAE581), a basic peptide nitrile, has been developed by Novartis and was found to be a potent and selective inhibitor of human CatK (IC50 = 0.0014 μM, compared to 2.9–28 μM for other cathepsins) [129]. Administered orally, was applied in osteoporosis treatment [130]. Another example is odanacatib (MK-0822) together with MK-1256 analogue [131,132]. Odanacatib inhibits CatK, B, L, and S with IC_50_ values of 0.2 nM, 1034 nM, 2995 nM, and 60 nM, respectively. CatK inhibition by MK-1256 was effective and selective among other cathepsins (IC50 = 0.62 nM, >1100-fold less potent against CatL and CatS *i.a.*). Incorporation of balicatib and odanacatib scaffolds was then evaluated by Burtoloso et al. in 2017 [133]. These authors developed new compounds with anti-trypanosomal activity, relevant for the discovery of further anti-chagasic agents. All depicted structures, and the inhibitory values are summarized in the Table 5.

For a broad review dealing with nitrile-based peptide inhibitors of cathepsins, supported by the knowledge of this inhibitory mechanism, we highlight the paper by Frizler et al. published in 2010 [134].

#### 3.3.3. Peptide Ketoheterocycles

(Keto)heterocyclic modifications of peptides were assessed in cathepsins inhibition at the beginning of 2000s. McGrath and colleagues synthesised a set of peptide α-ketoheterocyclic inhibitors, occupying the substrate recognition cleft of targeted cysteine protease CatK [135]. Molecular modeling supported by the crystallographic data revealed the reversible covalent bonding with Cys25 nucleophile. One of the most potent examples is the compound **4**, presented in the Table 6. *K*_i_ values calculated for **4** and CatK, B, L, and S were of 0.0029 nM, 0.64 nM, 0.02 nM, and 0.0066 nM, respectively.

Quibell group in 2004 and 2005 reported the synthesis and evaluation of *cis*-hexahydropyrrolo[3,2-*b*]pyrrol-3-one, and bicyclic peptidomimetic tetrahydrofuro[3,2-*b*]pyrrol-3-one and hexahydrofuro[3,2-*b*]pyridine-3-one derivatives as cysteinyl proteases inhibitors [136,137]. In their first paper, 5,5-bicyclic ketone-containing scaffold was depicted, underlining the valuable chiral stability. Kinetic analysis shown a significantly lower association with CatK of 6,5-analogues, and subsequently lower potency. Among all synthesised compounds, **10** was found to be the most effective and exhibited sub-micromolar potency in the cell-based assay (Table 6). This idea was further pursued and in 2005 another analogue, i.e., compound **22,** was described as an even more potent CatK inhibitor than those previously reported (Table 6).

Moreover, a summary of patents revealed in the field of cathepsin K inhibitors was published by Wijkmans and Gossen in 2011 [138].

## 4. Conclusions and Future Prospect

**Scheme 3:**
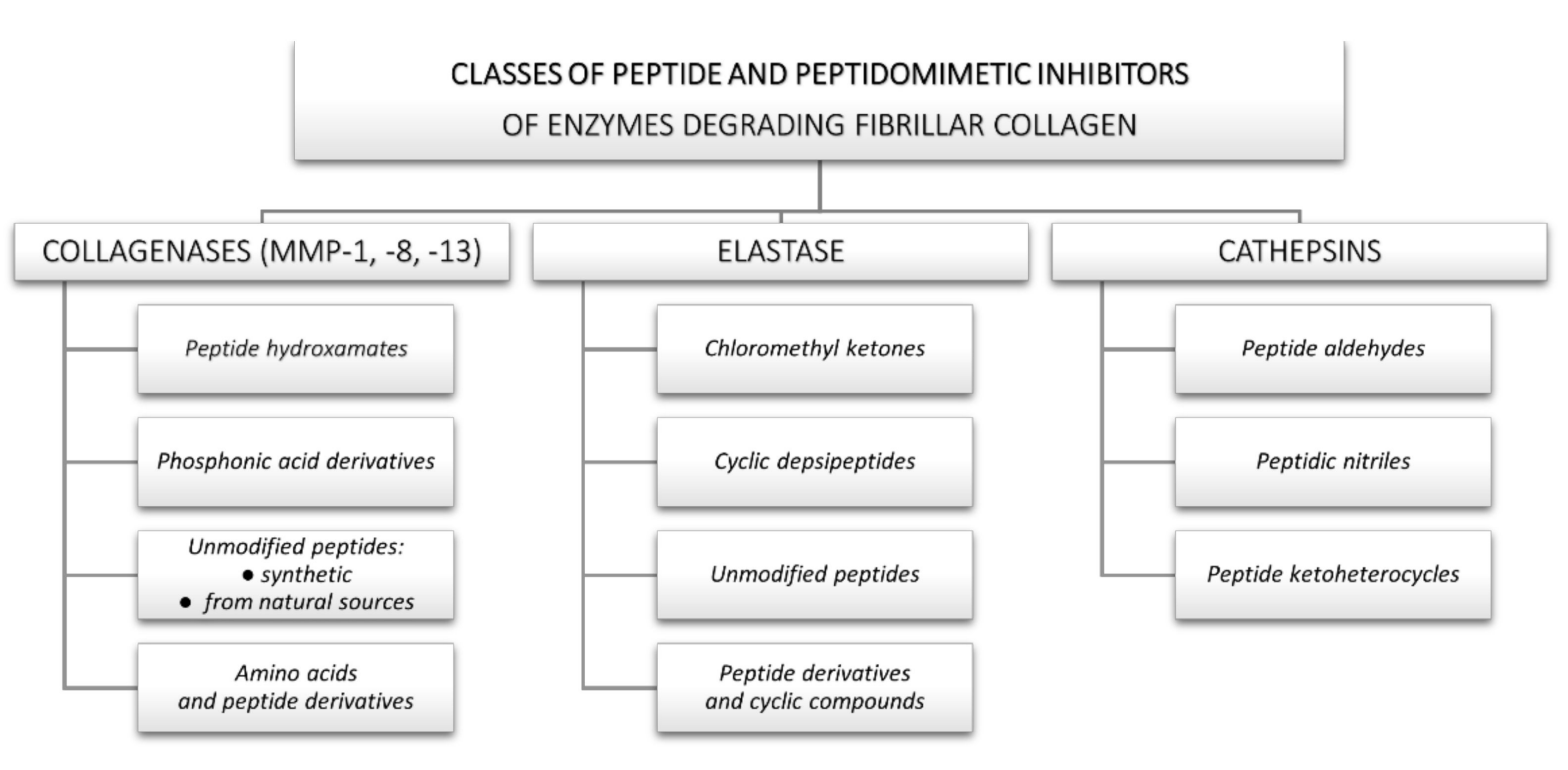
Scheme presenting all classes of inhibitors discussed in this review.

Collagen represents approximately one-third of all human body proteins, being the most abundant protein-like body component. The essential role played by collagen has an influence on many physiological, as well as pathological states. For example, dysfunctional collagen turnover and its increased degradation lead to wound healing disruption, skin photoaging, and loss of skin firmness and elasticity. Therefore, inhibitors of enzymes leading to the degradation of fibrillar collagen were in the spotlight for years, and still are the focus of many research projects. Healthy and younger-looking skin becomes increasingly significant targets, as they contribute to the wellness of each individual, as clearly shown by the critical growth of the skin-care products market observed recently. In this review, we describe a group of inhibitors of enzymes degrading fibrillar collagen, paying attention to peptides and peptidomimetics (Scheme 3 and Table 7). Additionally, a few valuable non-peptide molecules were mentioned, thereby providing a platform for further investigations and design of new active compounds. Prospectively, these peptides and peptidomimetics, together with their analogues, can be applicable as active ingredients in the cosmeceutical field.

## Figures and Tables

**Table 1:** Major enzymes involved in the extracellular degradation pathway of collagen [8,12,14,27,28,29,30,31,32,33,34,35,36,37,38,39,40,41].

Enzyme Family	Enzyme Common Name	Enzyme Classification or Abbreviation	Degraded Collagen Type	Type of Preferentially Degraded Fibrillar Collagen Type ^1^	Preferential Cleavage Site^3^
Matrix metalloproteinases	Collagenase-1	MMP-1	I, II, III, VII, VIII, X	I and III	Pro-neutral amino acid- 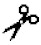 -Gly-Pro
	Collagenase-2	MMP-8	I–III, V, VII, VIII, X	I	
	Collagenase-3	MMP-13	II, III	II, III	
	Gelatinase A	MMP-2	IV–VI, X	n.a.	Pro-Gln-Gly- 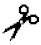 -Ile/Leu-Ala-Gly-Gln
	Gelatinase B	MMP-9	IV, V, VII, X, XIV	n.a.	
	Stromelysin-1	MMP-3	III	III (indirectly; by the MMP-1 activation)	Ser- 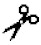 -Met
	Stromelysin-2	MMP-10	III–V	III (indirectly; by the MMP-1 activation)	
	Matrilysin	MMP-7	IV–X	n.a.	Ser- 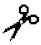 -Leu Ala- 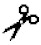 -Leu Tyr- 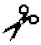 -Leu
	Metalloelastase	MMP-12	I, IV	n.a.	Pro-X-X- 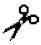 -Leu
	Matrilysin-2	MMP-26	IV	n.a.	Ser- 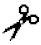 -Leu
	MT-MMP-1^2^	MMP-14	I, II, III	n.a.	Ser- 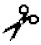 -Leu
	MT-MMP-3	MMP-16	III	n.a.	n.a.
Serine proteases	Elastase	-	I, III	I (independently and enhancing MMPs activity)	X- 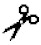 -Gly/Ala/Ser
	Trypsin-2	-	I, II, III	I (independently and enhancing MMPs activity)	Lys/Arg- 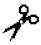 -X
Cysteine proteases	Cathepsin K	CatK	I, II	I, II	n.a.
	Cathepsin B	CatB	II	n.a.	n.a. (carboxypeptidase)
	Cathepsin L	CatL	I, II	I	Arg- 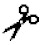 -X
	Cathepsin S	CatS	I	I	n.a.

^1^ n.a.: no data available. ^2^ MT-MMP: membrane-bound MMP. ^3^ 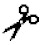: cleaved bond.

**Table 2:** Selectivity of human collagenases inhibition, among available inhibitors of metalloproteases (by Moore and Spilburg) [63].

Trade Name	IUPAC or PubChem Name	Structure	% of Inhibition
Amastatin	(2*S*)-2-[(2*S*)-2-[(2*S*)-2-[(2*S*,3*R*)-3-Amino-2-hydroxy-5-methylhexanoyl]amino]-3-methylbutanoyl]amino]-3-methylbutanoyl]amino]butanedioic acid	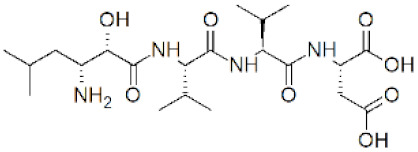	0
Captopril	(2*S*)-1-[(2*S*)-2-methyl-3-sulfanylpropanoyl]pyrrolidine-2-carboxylic acid	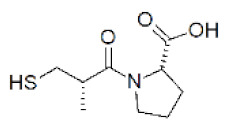	0
Phosphoramidon	(2*S*)-2-[(2*S*)-2-[hydroxy-[(2*S*,3*R*,4*R*,5*R*,6*S*)-3,4,5-trihydroxy-6-methyloxan-2-yl]oxyphosphoryl]amino]-4-methylpentanoyl]amino]-3-(1*H*-indol-3-yl)propanoic acid *N*-[*N*-[(6-deoxy-α-L-mannoopyranosyl)oxy]hydroxyphosphinyl]-L-leucyl]-L-tryptophan	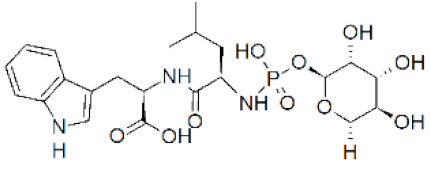	0
Zincov	2-(N-hydroxycarboxamido)-4-methylpentanoyl-alanyl-glycinamide	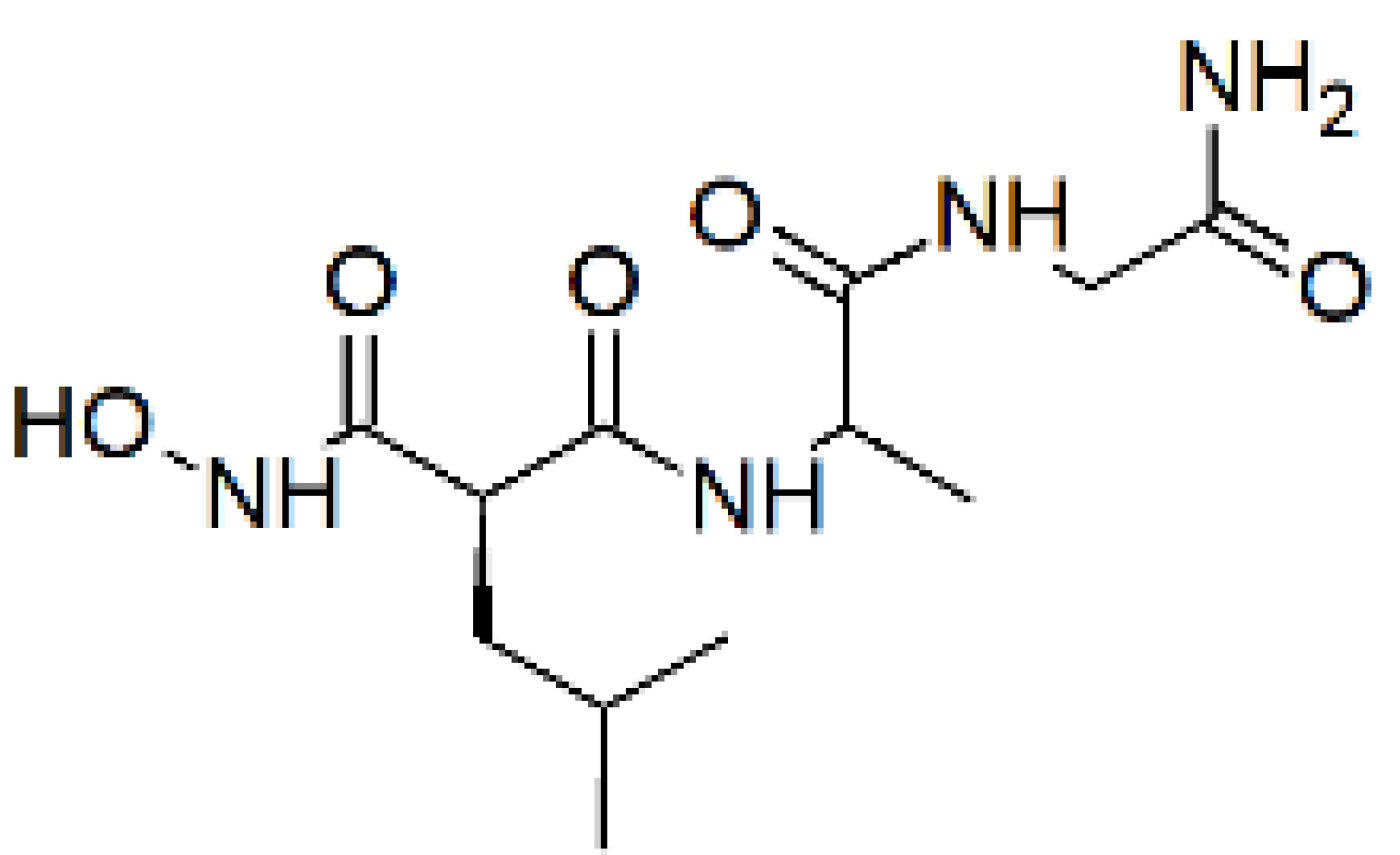	27

**Table 3:** Mycosporine-like amino acids (MAA) from marine sources and two standard inhibitors, tested in the collagenase inhibition assay described by Hartmann et al. [93].

Name	IC_50_ Value
Shinorine	104.0 ± 3.7
Porphyra	105.9 ± 2.3
Palythine	158.9 ± 3.2
Phosphoramidone ^1^	18.8 ± 1.6
1,10-phenanthroline ^1^	238.1 ± 3.4

^1^ Standard inhibitor.

**Table 4:** Lipopeptides reported in Toth et al. [109] All structures are based on X-AAPV-Y fragment, with proper N- and C-terminal modifications (X and Y, respectively).

Name	Structure
**1a**	CH_3_(CH_2_)_7_CH=CH(CH_2_)_7_CO-AAPV-OH
**1b**	(CH_3_)_3_COCO-DL-NH[CH(CH_2_)_11_CH_3_]-AAPV-OH
**1c**	(CH_3_)_3_COCO{DL-NH[CH(CH_2_)_11_CH_3_]CO}_2_-AAPV-OH
**1d**	(CH_3_)_3_COCO{DL-NH[CH(CH_2_)_11_CH_3_]CO}_3_-AAPV-OH
**1e**	(CH_3_)_3_COCO{DL-NH[CH(CH_2_)_11_CH_3_]CO}_3_-AAPV-OCH_3_

**Table 5:** Structures and IC_50_ values obtained for peptidic nitriles, selective inhibitors of cathepsin K.

Compound	Structure	IC_50_ Value for CatK	Reference
Balicatib (AAE581)	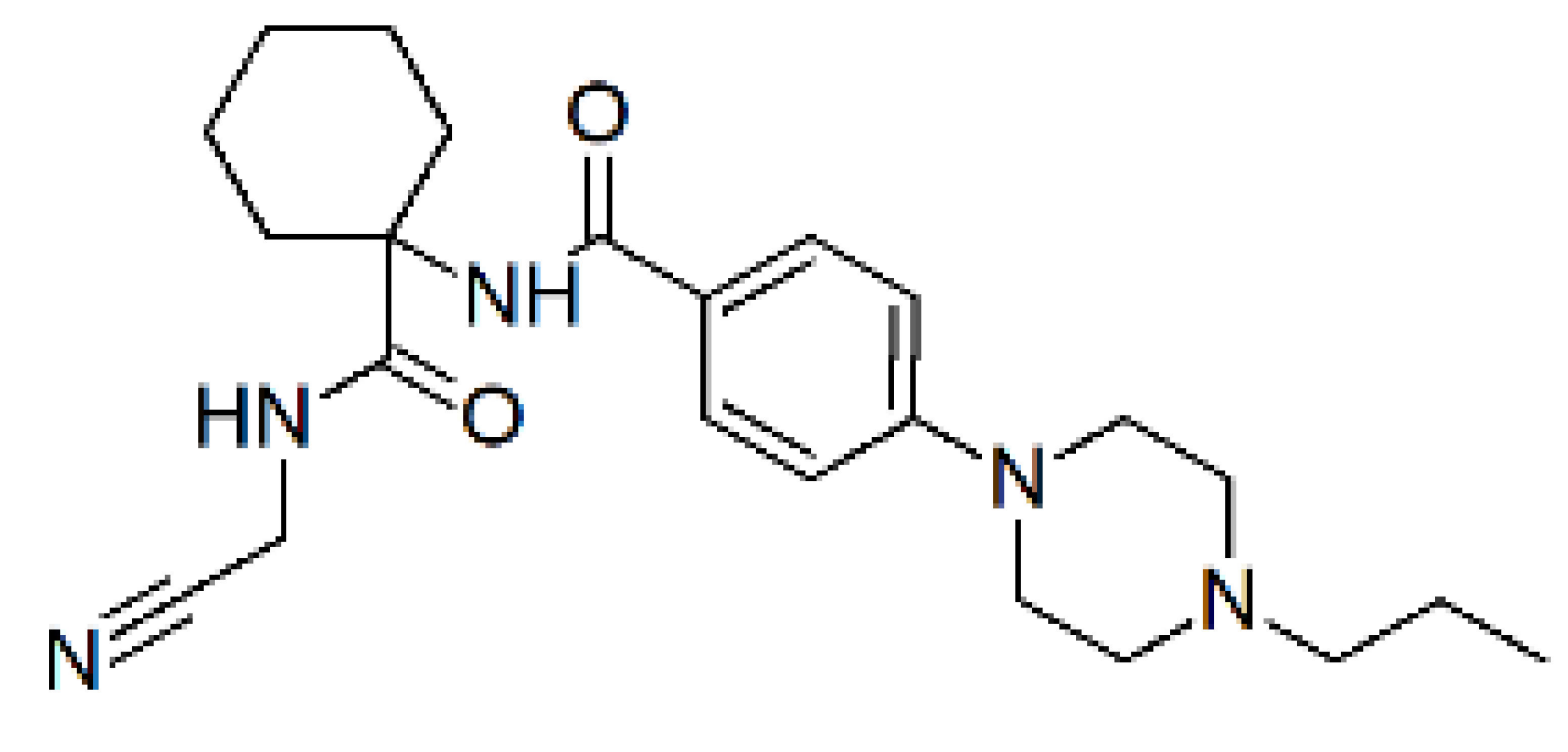	0.0014 μM	[129]
Odanacatib (MK 0822)	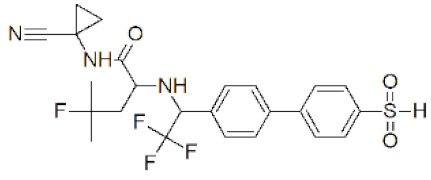	0.2 nM	[131]
MK-1256	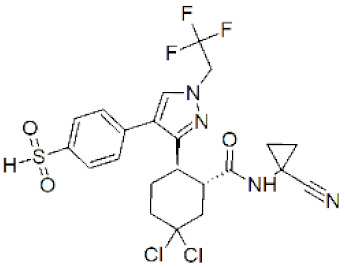	0.62 nM	[132]

**Table 6:** Structures and *K*_i_ values in nM obtained for heterocyclic peptidomimetics, synthesised and evaluated against various cathepsins. Results for CatK, L, and S are evidenced.

Compound	Structure	Cathepsin K *K*_i_ [nM]	Cathepsin L *K*_i_ [nM]	Cathepsin S *K*_i_ [nM]	Reference
**4**	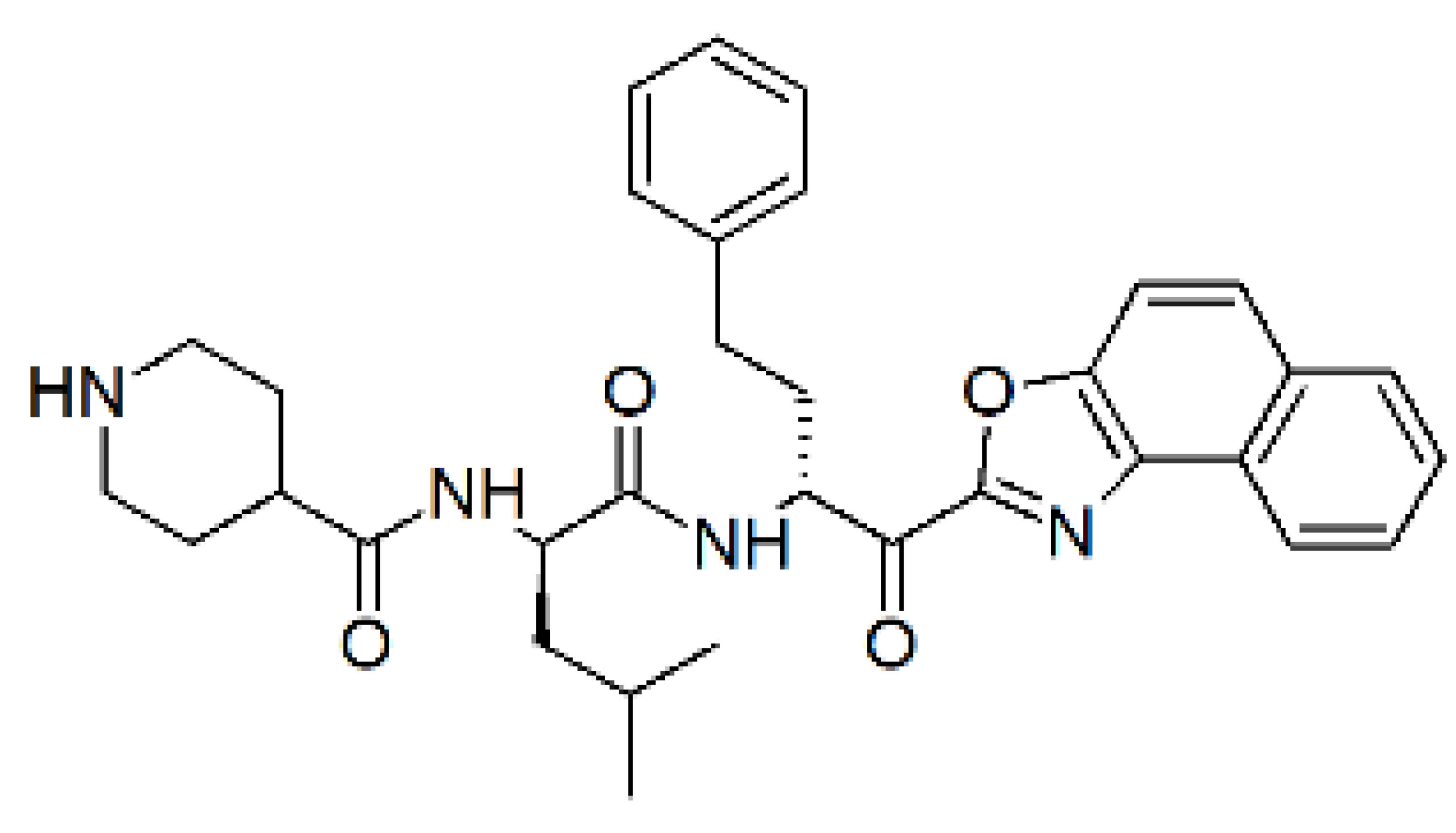	0.0029	0.02	0.0066	[135]
**10**	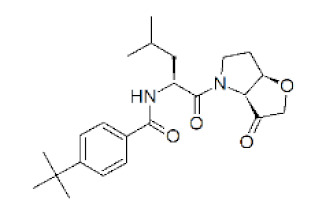	87.4 +/− 0.8	>25,000	>41,000	[136]
**22**	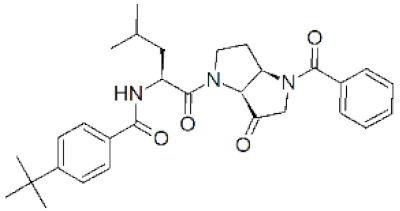	10.1 +/− 6.7	>3500	>4500	[137]

**Table 7:** Summary of unmodified peptide sequences exhibiting collagenases and elastases inhibition, together with peptide aldehydes inhibiting cathepsins, evaluated in this review.

Peptide Symbol	Enzyme Class	Sequence of an Inhibitor	Reference
**A**	Collagenases	ACYCRIPACIAGERRYGTCIYQGRLWAFCC	[81]
**B**		NLDVLEVF	[91]
**C**		CSCSDMTDKECLYFCMSEMS	[84]
**D**		PRCBCGE	[85]
**E**		CDCAPV	[86]
**F**		GGDEDDLSEEDLQFAERYLRSYYHPT DLSEEDLQFAERYLRSYYHPT	[87]
**G**			
**H**		GLAGQRGIVGLOGQRGERGFOGLOGRS GLOGERGRTGPAGAAGARGNDGQOGPA	[89]
**I**			
	
**J**		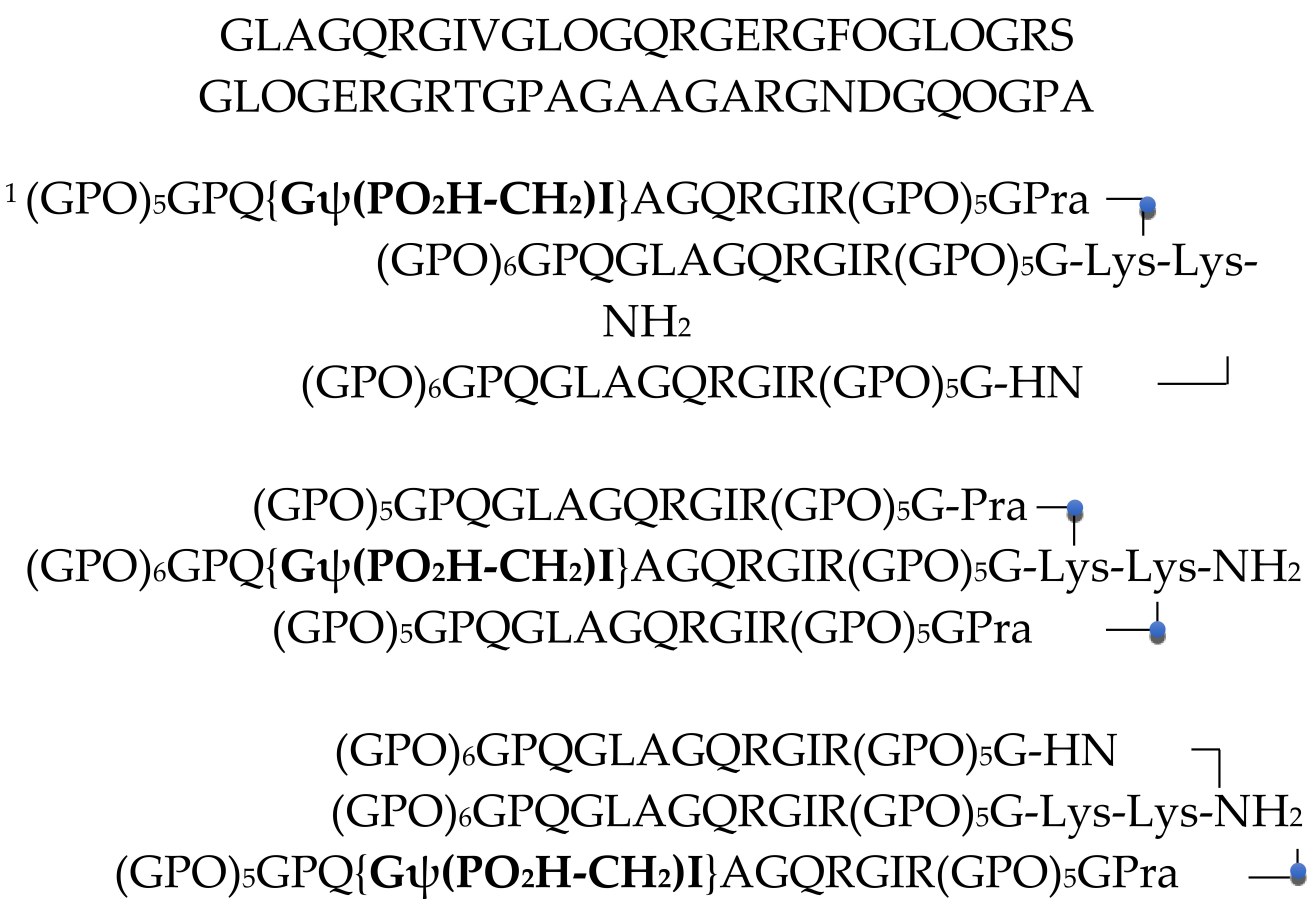	[90]

**K**			

**L**			
**M**	Elastase	(CH_3_)_3_COCO{DL-NH[CH(CH_2_)_11_CH_3_]CO}_3_-AAPV-OH	[109]
**N**		PMTLEYR	[110]
**O**		MGWCTASVPPQCYG MGWCTASVPPQCYG(GA)_7_	[112]
**P**			
**R**		KDRCLLPKVTGPCKASLTRYYYDKDTKACVEFIYGGCRGNRNNFKQKDECEKACTDH	[114]
**S**		OT-dehydroxyT-AA_1_-F-AA_2_-V ^2^	[115,116]
**T**	Cathepsins	Ac-LLR-CHO	[124]
**U**		N-[Nα-carbonyl-RVR(aldehyde)]-F	[125]
**V**		Ac-LLnorL-CHO Ac-LLM-CHO	[126]
**X**			
**Y**		Cbz-LLL-CHO	[127]
**Z**		Cbz-FL-COCHO	[128]

^1^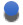 – indicates the sites of click ligation; O – Hyp. ^2^ AA_1_, AA_2_—non-abbreviated, unnatural amino acids. For cyclic and detailed structures, please see the references.

## Data Availability

No new data were created or analysed in this study. Data sharing is not applicable to this article.
